# GlyStruct: glycation prediction using structural properties of amino acid residues

**DOI:** 10.1186/s12859-018-2547-x

**Published:** 2019-02-04

**Authors:** Hamendra Manhar Reddy, Alok Sharma, Abdollah Dehzangi, Daichi Shigemizu, Abel Avitesh Chandra, Tatushiko Tsunoda

**Affiliations:** 1 School of Engineering & Physics, University of the South Pacific, Suva, Fiji; 2 Laboratory for Medical Science Mathematics, RIKEN Center for Integrative Medical Sciences, Tokyo, Japan; 3 Institute for Integrated and Intelligent Systems, Griffith University, Brisbane, Australia; 4 CREST, JST, Tokyo, Japan; 5 Department of Computer Science, Morgan State University, Baltimore, MD USA; 6 Division of Genomic Medicine, Medical Genome Center, National Center for Geriatrics and Gerontology, Obu, Aichi Japan; 7 Department of Medical Science Mathematics, Medical Research Institute, Tokyo Medical and Dental University, Tokyo, Japan

**Keywords:** Post-translational modification, Lysine glycation, Protein sequences, Amino acids, Prediction, Support vector machine

## Abstract

**Background:**

Glycation is a one of the post-translational modifications (PTM) where sugar molecules and residues in protein sequences are covalently bonded. It has become one of the clinically important PTM in recent times attributed to many chronic and age related complications. Being a non-enzymatic reaction, it is a great challenge when it comes to its prediction due to the lack of significant bias in the sequence motifs.

**Results:**

We developed a classifier, *GlyStruct* based on support vector machine, to predict glycated and non-glycated lysine residues using structural properties of amino acid residues. The features used were secondary structure, accessible surface area and the local backbone torsion angles. For this work, a benchmark dataset was extracted containing 235 glycated and 303 non-glycated lysine residues. *GlyStruct* demonstrated improved performance of approximately 10% in comparison to benchmark method of *Gly-PseAAC.* The performance for *GlyStruct* on the metrics, sensitivity, specificity, accuracy and Mathew’s correlation coefficient were 0.7013, 0.7989, 0.7562, and 0.5065, respectively for 10-fold cross-validation.

**Conclusion:**

Glycation has emerged to be one of the clinically important PTM of proteins in recent times. Therefore, the development of computational tools become necessary to predict glycation, which could help medical professionals administer drugs and manage patients more effectively. The proposed predictor manages to classify glycated and non-glycated lysine residues with promising results consistently on various cross-validation schemes and outperforms other state of the art methods.

**Electronic supplementary material:**

The online version of this article (10.1186/s12859-018-2547-x) contains supplementary material, which is available to authorized users.

## Background

Post-translational modifications (PTM) of protein occur when there is a covalent alteration to protein backbones and side chains that increase proteome complexities. PTMs are generally mediated by enzymatic activity that occur at selected sites along amino acid side chains after its translation by ribosome is complete [1, 2]. These modifications provide important insight into various cellular functions and biological processes of proteins such as cellular dynamics and elasticity [3, 4]. There are many important PTMs with significant biological impact such as acetylation, carbonylation, glycosylation, glycation, methylation, nitrosylation, phosphorylation, sumoylation, succinylation, and ubiquitylation to name a few [5–10].

Of lately, glycation has emerged to be of significant clinical relevance attributed to a correlation with increased blood glucose concentration [11, 12], and metabolic morbidity detection [13]. This biochemistry involves a complex multi-step site modification process between reducing sugars and amino acid groups located in lysine (K) and arginine (R) residues, or in the *N*-terminal position to form Amadori adduct [14, 15]. The Amadori adduct further reacts to form advanced glycation endproducts (AGEs). With aging, AGEs accumulate and alters the tissue protein structure, function and turnover. If untreated, AGEs can lead to chronic complications of diabetes mellitus and neurodegenerative changes such as Alzheimer’s disease and amyotrophic lateral sclerosis [16–24]. Moreover, correlations have been established between levels of AGEs and diabetes with its related complications [7, 20, 24–26] in aging *Homo sapiens.* Glycation being a non-enzymatic reaction presents a great challenge in detection due to the motifs having greater levels of entropy compared to other PTMs. Conversely, enzymatic reaction is characterized by a more specific reaction and often has more biased sequence motif [27, 28].

In clinical methods, PTMs are identified in wet labs by observing this modification using methods such as mass spectrometry and immunofluorescence, and stored in online databanks such as dbPTM, CPLM and PLMD [1, 29–31]. Despite PTM being an important area for morbidity detection and genetics, clinical approaches face great limitation due to the plethora of protein sequences in existence in data repositories [32], high costs, and time-consuming process of biochemical experimentations in wet-labs [3]. Hence, data scientists have been exhorted to actively pursue the development of computational tools to provide cost-effective solutions [3, 33–35]. This has led to an evolution of data mining in medicine, especially in the area of proteomics [36–38]. A concerted international effort has seen large dataset being actively developed to study and predict site-specific protein modification [31, 39].

While clinical importance of glycation is obvious, on the contrary however, few predictors have been proposed for this type of PTM. The earliest predictor, *GlyNN* [27] was developed using artificial neural network involving a dataset of only 89 glycated and 126 non-glycated lysines residues from a set of 20 proteins. *PreGly* predictor by Liu et al. [40] built on the same dataset as [27] used composition of 푘-spaced amino acid pairs (CKSAAP) for extracting features from protein sequences. *GlyNN* achieved the sensitivity, specificity, accuracy and Mathew’s correlation constant (MCC) of 0.7865, 0.8015, 0.795 and 0.58, respectively, while *PreGly* achieved for the same metrics, 0.7106, 0.9585, 0.8551 and 0.7 respectively. *Gly-PseAAC* developed by Xu et al. [28] used the recently updated dataset from CPLM databank consisting 223 glycated and 446 non-glycated residues. They have considered features from position-specific amino acid propensity (PSAAP) scheme. More recently, Zhao et al. proposed *Glypre* predictor [41] using a combination of features like position conservation, amino acid index and CKSAAP. In addition, Islam et al. [42] investigated an even larger set of features that included propensity based features, amino acid composition, physicochemical features and secondary structure motifs for their predictor *iProtGly-SS*. The results obtained by [28] on the on the recent dataset is low with sensitivity at 0.5748 and specificity at 0.7430. Furthermore, *Glypre* and *iProtGly-SS* reported performance on the two datasets from Johansen [27] and Xu et al. [28] but applied various filtering techniques to overcome the problem of data imbalance between negative and positive instances. *Glypre* excels with dataset from [27], but it achieved sensitivity at only 0.5747 while demonstrating high specificity of 0.9078 on the larger dataset from [28]. On the same new dataset, *iProtGly-SS* predictor, manages higher sensitivity of 0.9238. However, their specificity reached maximum of 0.6009. All comparison are made for 10-fold validations since they are generally higher. For clinical use, however, glycation needs a more robust prediction of both instances of glycated and non-glycated lysines. Therefore, there is an opportunity to explore alternative methods for more robustness and any slight improvement in prediction provides a valuable resource to the community [43].

To predict glycation sites with high accuracy and to address the shortcoming of those previous studies, we introduce a new machine learning method called *GlyStruct* to predict glycation of lysines. To develop *GlyStruct* predictor, we incorporated structural information extracted from the predicted local structure of protein sequences as our input feature set and employed Support Vector Machine (SVM) as a classifier [44, 45]. Our achieved results demonstrate that *GlyStruct* is capable of predicting both, the glycated and non-glycated lysine residues better than previously proposed method found in the literature for this task. Using *GlyStruct*, we achieved 0.7013, 0.7989, 0.7562, and 0.5065 for sensitivity, specificity, accuracy and Mathew’s correlation coefficient, respectively for the 10-fold cross validation.

## Methods and materials

To build our predictor model, benchmark dataset was curated from the online databanks. Following the standard methodology in bioinformatics [3], the dataset was then formulated to make it suitable for training classifiers and an appropriate cross-validation scheme was used to objectively evaluate the accuracy of the predictor.

This section describes the proposed method and benchmark dataset used in this study.

### Benchmark dataset

The dataset for glycation was obtained from publically available and widely used CPLM database [30] (available http://cplm.biocuckoo.org/) that was curated from comprehensive clinical and in vitro studies [43]. The benchmark dataset we retrieved was filtered for redundant sequences with a threshold of 30% for pairwise sequence identity. The final dataset consisted of 1753 lysine sites in total found in 55 proteins. Among them, 235 lysines are glycated and 1518 are non-glycated sites. The primary sequences used to build GlyStruct are included in supplement as the Additional file 1.

### Feature extraction

The secondary structure features reveal intrinsic information regarding the characteristics of a protein sequence. In this study, we considered three attributes that formulate the local structure of protein namely, the secondary structure, local backbone torsion angles, and accessible surface area (ASA). The prediction of those attributes was carried out using the SPIDER2 toolbox [46]. The SPIDER2 toolbox demonstrated promising result predicting these attributes compared to other methods found in the literature for predicting secondary structure [47, 48], backbone angles [49, 50], and accessible surface area [46, 49, 51] of amino acids. Predicted results using SPIDER2 has been used in different studies and demonstrated promising results [52–54]. The following describes the features integrated in this work:

**Accessible Surface Area (ASA)** provides an estimate surface area of a particular amino acid reachable by a solvent situated in the protein’s three-dimensional configuration [55, 56]. The predicted values of ASA for individual amino acids hence provides essential information of how it locally interacts with other amino acids to build global protein structure.

**Secondary structure** provides insight into the local three-dimensional structure within protein sequence where each amino acid can be discriminated based on the three defined local backbone folding patterns corresponding to a polypeptide. These are helix (*ph*), strand (*pe*) and coil (*pc*) motifs. Information from the secondary structure can contribute constructively to the general three-dimensional configuration of the polypeptide and the affinity for PTM of lysine residues [54, 57]. Given a protein sequence, SPIDER2 produces a *L* × 3 matrix containing the predicted secondary structure, which we call *SSpre*. *L* represents the length of a protein sequence and columns represent the transitional probabilities of each amino acid conforming to the three secondary structures.

**Fig. 1:**
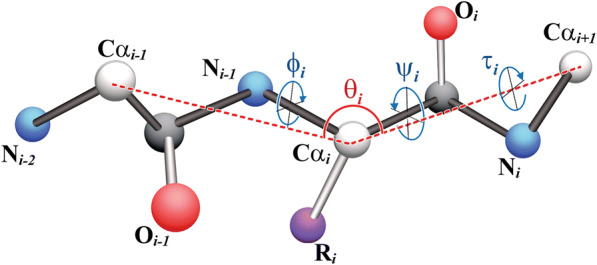
Local backbone torsion angles of polypeptide showing relevant bonds

**Local Backbone angles** refer to the torsion angles between neighbouring amino acids that provide backbone conformations (local structure) of a polypeptide. They complement the information provided by *ASA* and the secondary structure predictions (*SSpre)* [50] of amino acids. The predicted backbone torsion angles, *ϕ, ψ, θ, τ*, represent the interaction of local amino acid along the protein backbone [54, 58, 59] as shown in Fig. 1 [60]. *Φ* and *ψ* demonstrate the torsion angles among the molecules inside one single amino acid with respect the neighboring molecules. On the other hand, *θ* and *τ* demonstrates torsion angles between Alpha Carbons (Cα) in neighboring amino acids [49]. In fact, *θ* determines torsion angles between three neighboring Cα and Cα_i − 1_ − Cα_i_ − Cα_i + 1_ while τ determines the torsion angles between two neighboring Cα and Cα_i_ − Cα_i + 1_. While secondary structure provides the general elucidation around sections of peptide, local backbone angles provide elaboration of structure within the locality of PTM points, the latter being lysine residue in this case.

### Feature vector construction

Protein sequences are of varying lengths and cannot be used directly in classification. Classifiers require dataset of fixed length [61] therefore we employed a widely used method of truncating the protein sequence into fixed length peptide segments [54, 57, 62–66] proposed by Chou [67, 68].

**Fig. 2:**
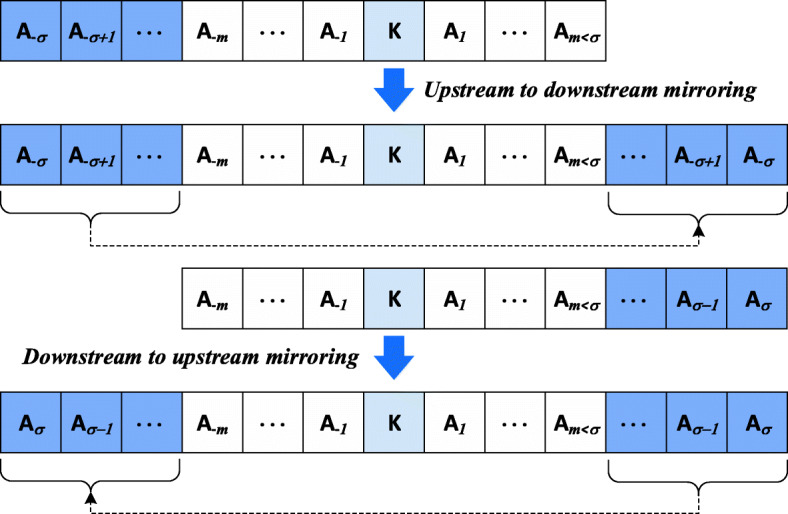
Illustration of selecting window size to obtain feature vector for training and testing classifier

We selected the peptide segment by sliding a window of size δ amino acids on the primary sequence taking the flanking upstream and downstream sequence of amino acids on each side of lysine residue *K,* with a flank of size σ as shown in Fig. 2. Segment window size of δ = 13 consistently produced optimized results after testing out all window sizes from δ = 3 to δ = 39. As a result, the flank size was determined as σ = 6.

**Fig. 3:**
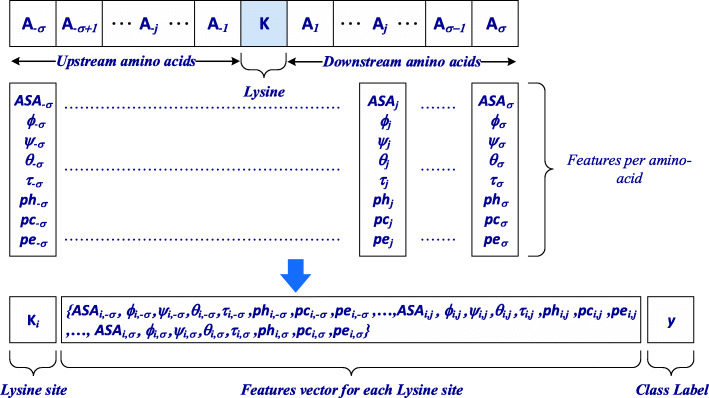
mirroring used to obtain consistent feature vector size for instances where lysine sites were located towards beginning or end of protein sequence leaving insufficient flanking amino acids for feature vector

If a lysine residue flank (either upstream or downstream) did not contain enough amino acids to create a consistent flank size specified by σ, the void portion was filled using mirror effect [54, 62, 69] (Fig. 3). The segment sequence ${S}_{{\mathrm{K}}_{\mathrm{i}}}$ comprising lysine residue *K* with flanking upstream and downstream amino acids *A*_i_ can be expressed as follows:

1 $$ {S}_{K_i}=\left\{{A}_{-6},{A}_{-5},\dots, {A}_{-2},{A}_{-1},{K}_i,{A}_1,{A}_2,\dots, {A}_5,{A}_6\right\} $$

where *A*_*j*_ (for 1 ≤ j ≤ 6) denotes downstream amino acids of the lysine; *A*_−*j*_ (for 1 ≤ j ≤ 6) the upstream amino acids of the lysine; and *K*_i_, the lysine residue itself at *i*^th^ position in the protein sequence. The size of ${S}_{{\mathrm{K}}_{\mathrm{i}}}$ is 13 amino acids that includes the lysine residue *K* and the 6 amino acids on each side. The segment sequence ${S}_{{\mathrm{K}}_{\mathrm{i}}}$ has a class label *y* corresponding to its lysine residue, which can be written as *y* = {0, 1}. For the case when ${S}_{{\mathrm{K}}_{\mathrm{i}}}$ describes a glycated lysine residue, the label is *y* = 1 and a non-glycated lysine residue is represented by *y* = 0. In addition, each amino acid *A*_*j*_ and *A*_−*j*_ is designated by the structural features *F*_*i*_ as expressed in Eq. 2.

2 $$ {F}_i=\left\{ ASA,\phi, \psi, \theta, \tau, ph, pe, pc\right\} $$

The features set *F*_*i*_ presented in Eq. (2) for each amino acid is an 8-dimensional vector which is concatenated with the features of the whole segment (13 amino acid) producing a 104-dimensional vector. The appropriate class label (*y* = 1 and *y* = 0) for each instance of the lysine residue is considered for developing the classifier.

### Classification engine

SVM works by establishing an optimal hyperplane between classes and extends to patterns that are not linearly separable by using kernel functions. If the dimensionality of feature vectors is very high, then dimensionality reduction techniques can be employed before SVM application [70–79].

In SVM algorithm (Eq. 3), the margin between hyperplanes needs to be minimized, which represent boundaries between classes (of glycated and non-glycated lysines). If the boundaries are non-linear, kernels functions are used [80]. The kernel functions can be non-linear such as radial basis function (RBF), polynomial and sigmoid. In this work, we designed our *GlyStruct* predictor using SVM with a polynomial kernel function to find a margin between glycated and non-glycated lysine residues. To predict the class label *y*^′^ of an unknown lysine residue with *x*^′^ feature vector the following function is used

3 $$ {y}^{\prime }=\operatorname{sign}\left({\sum}_{\mathrm{i}=1}^n{\upalpha}_{\mathrm{i}}{y}_{\mathrm{i}}\upkappa \left({x}_{\mathrm{i}},{x}^{\prime}\right)+\upbeta \right) $$

where α_i_ are adjustable weights, *n* is the number of samples, *β* is representing the bias and *κ*(.) is the kernel function.

We designed our classifier using *libsvm* [81], a publicly available and widely used SVM tool, and also accessible on WEKA platform [82]. Tuning parameters were obtained using grid-search where *C* = 512, and γ = 0.03125. We used polynomial learning because it provided better results given by (x_i_^T^x_j_ + C_0_)^d^ where we used C_0_ = 0 and degree of polynomial *d* was taken as 3.

## Results and discussion

### Prediction metrics

The true positive rate or sensitivity is an important performance indicator of the ability of the classifier to predict the glycated lysine residues correctly. The metric varies between 0, (that is classifier is totally inaccurate) and 1 (signifying the classifier is totally accurate). Hence the higher the true positive rate, the better the classifier performance is at detecting the glycated lysine residue. Sensitivity is given by

4 $$ \mathrm{Sensitivity}=\frac{\mathrm{TP}}{\mathrm{TP}+\mathrm{FN}} $$

where *TP* (true positive) denotes the number of correctly identified glycated instances from the test set, and *FN* (false negative) denotes the number of incorrectly classified glycated sites.

The true negative rate or specificity is the ability of the classifier to identify negative (non-glycated) instances. This metric also has a range between a value of 0 (totally incorrect) and a 1 (totally correct) in classifying the non-glycated lysine residues. *TN* (true negative) denotes the number of non-glycated instances identified and *FP* (false positive) denote the non-glycated sites identified as glycated.

5 $$ \mathrm{Specificity}=\frac{\mathrm{TN}}{\mathrm{TN}+\mathrm{FP}} $$

Accuracy (Acc) metric is measured as the total number of both glycated and non-glycated lysine residues correctly classified over the total number of test instances (*N*). This metric also takes on values between 0 (totally inaccurate) and a 1 (totally accurate).

6 $$ \mathrm{Acc}=\frac{\mathrm{TP}+\mathrm{TN}}{\mathrm{N}} $$

Mathew’s correlation coefficient (MCC) metric essentially measures the quality of classification for a classifier. This metric varies between – 1 (total misclassification), 0 (no better than random prediction), and 1 (perfect prediction of test instances).

7 $$ \mathrm{MCC}=\frac{\left(\mathrm{TN}\times \mathrm{TP}\right)-\left(\mathrm{FN}\times \mathrm{FP}\right)}{\sqrt{\left(\mathrm{TP}+\mathrm{FP}\right)\left(\mathrm{TP}+\mathrm{FN}\right)\left(\mathrm{TN}+\mathrm{FP}\right)\left(\mathrm{TN}+\mathrm{FN}\right)}} $$

The best performing predictor will be the one scoring the highest in majority of the four metrics.

### Evaluation methods

The effectiveness of any classifier is measured using cross-validation methods. The three most widely used cross-validation schemes across the literature are independent dataset, *k*-fold and jackknife [83, 84]. Since the dataset for glycation in the curated protein sequences is limited, it was not practical to obtain additional data to run independent test validation.

The *k*-fold cross-validation procedure is carried out by first partitioning the total benchmark dataset into *k* roughly equal folds. Then one fold is held as a test set and the remaining *k* − 1 folds are used to train the classifier and a model is constructed. Using the constructed model and the test dataset that was held out, all prediction metrics are computed. This procedure is repeated *k* times as per the fold number chosen to obtain the average of the performance metrics.

Jackknife process can be viewed as a special instance of *k*-fold when *k* is *n*-1, where *n* is the number of samples. While the jackknife method is recognized as the least arbitrary that outputs unique results on the given benchmark dataset [85], the *k*-fold method offers an advantage whereby all instances or observations in the dataset can be used in both the training and test phases.

To evaluate our *GlyStruct* predictor, we carried out *k*-fold cross validation for 6-, 8- and 10-folds and jackknife test which is a common practice [28, 38, 40, 54, 62].

### Sample filtering

The dataset for our study comprised 235 glycated and 1518 non-glycated lysine residues obtained from 55 protein sequences, which results in a highly imbalanced data between positive (glycated) and negative (non-glycated) sets with a ratio of over 1:6. While it is a natural phenomenon in the biological sense, it creates a strong bias to the negative (or non-glycated) class if the dataset is used as is to train virtually any classifier. Therefore, we used *k*–nearest neighbor (*k*NN) filter to resolve the imbalance in dataset, similar to the approach taken by Jia et al. [62] and López et al. [54]. Subsequently, the *k*NN cleaning treatment with a *k* value of 16 brought down the number of negative samples to 303. In other words, the cleaning treatment reduced the negative samples (non-glycated sites) by removing those samples, which were within the 16 neighbors of a positive sample (glycated site) to achieve 235 positive samples and 303 negative samples.

### Comparison with benchmark prediction methods

**Fig. 4:**
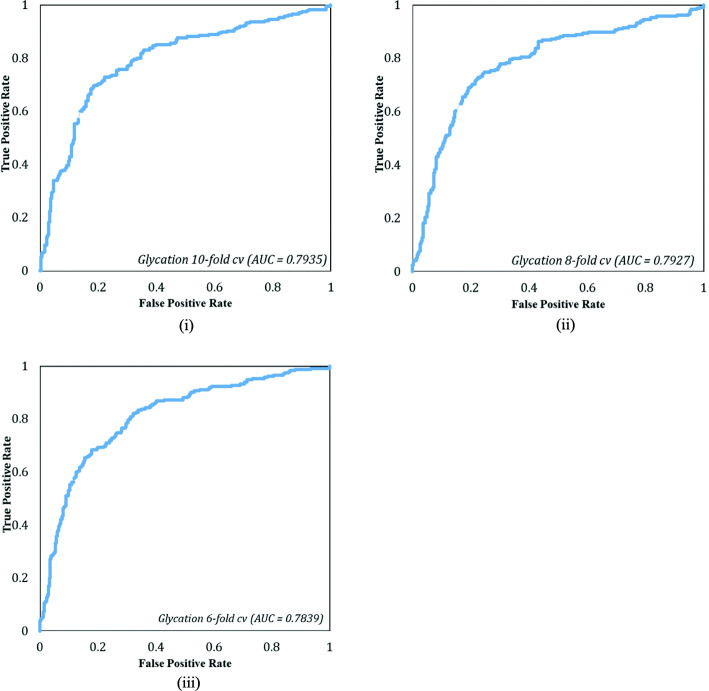
AUC curves of *GlyStruct* corresponding to (**i**) 10-, (**ii**) 8-, and (**iii**) 6-fold cross-validations

We obtained promising results from the *GlyStruct* predictor presented in Table 1. For statistical stability, we took an average of 50 runs for each cross-validation fold. We obtained the highest sensitivity 0.7059 for 8-fold cross-validation while other folds recorded marginally lower sensitivity within 1 %. We also achieved high specificity at 0.7989 for 10-fold with a deviation of half percent for other folds. The best values of accuracy and MCC were 0.7562, and 0.5065 respectively (both in 10-fold). The 6-fold results yielded slightly lower than other folds with 0.6984, 0.795, 0.7528 and 0.4983 for sensitivity, specificity, accuracy and MCC, respectively. The AUCs were 0.7935, 0.7927 and 0.7839 (Fig. 4), for 10-, 8- and 6-folds, respectively. Mathew’s correlation coefficient (MCC) is around 0.5 for each fold indicating that the predictor performance is promising for glycation prediction. Jackknife procedure yielded highest sensitivity of 0.7404 and, specificity, accuracy, and MCC were 0.7793, 0.7622 and 0.5186 respectively.

**Table 1:** Performance evaluation of *GlyStruct* and compared with other existing method

Method	Sensitivity (%)	Specificity (%)	Accuracy (%)	MCC
GlyStruct (10-Fold)	0.7013	0.7989	0.7562	0.5065
GlyStruct (8-Fold)	0.7059	0.7952	0.7562	0.5059
GlyStruct (6-Fold)	0.6984	0.7950	0.7528	0.4983
GlyStruct LOO	0.7404	0.7793	0.7622	0.5186
Gly-PseAAC^a^ (10-Fold)	0.6845	0.6745	0.6784	0.3587
Gly-PseAAC^a^ (8-Fold)	0.6768	0.6751	0.6784	0.3514
Gly-PseAAC^a^ (6-Fold)	0.6830	0.6776	0.6785	0.3579
Gly-PseAAC^b^ LOO	0.5874	0.7399	0.6891	0.3198

^a^*Gly-PseAAC* predictor performance on our dataset

^b^as reported in [28] for *Gly-PseAAC*

We compared our results to the state of the art of bioinformatics study on glycation *Gly-PseACC* [28], which was the only predictor that had the webserver available for testing our dataset.

The dataset retrieved by *Gly-PseAAC* authors from CPLM database is larger than *GlyNN* and *PreGly*, which consisted 223 positive and 446 negative samples filtered from 72 protein sequences with 40% pairwise sequence identity. Their dataset is slightly different (by approximately 5% for positive samples) from the *GlyStruct* dataset of 235 positive and 303 negative samples from 55 proteins obtained after filtering with a threshold of 30% pairwise sequence identity. Therefore, to compare the performance of *Gly-PseAAC* webserver, we uploaded our dataset manually to the *Gly-PseAAC* webserver by creating a *FASTA* file format. The performance results we obtained from the webserver are presented together with the *GlyStruct* performance in Table 1.

There was a notable increase in the sensitivity of 0.6845 for *Gly-PseAAC* method with our dataset from their reported value of 0.5748 for 10-fold. We anticipate that most of the protein sequences we tested on their webserver may have been used in training their model primarily because of the limited datasets available publically in databanks. In addition, the *Gly-PseAAC* server has been tuned to a threshold probability of 0.35 allowing higher misclassification of negative samples leading to very high fall out or false positive rate averaging 32% for the three *k*-fold validation schemes. High false positive rate may have a serious bearing on the clinical significance in terms of better morbidity detection. In contrast, the specificity of *Gly-PseAAC* for 10-fold was reduced to 0.6745 from the reported 0.8017 and MCC was also slightly lower on our dataset (0.3587 compared to their reported 0.38). The accuracy was also slightly lower (0.6784) compared to their reported results (0.6812). In order to show the significance of the achieved results for *GlyStruct*, pairwise *t*-test was conducted. The *p*-values obtained were 0.025, 0.019, 0.025 for 10-, 8- and 6-folds respectively. These *p*-values are less than 0.05, which demonstrates that improvement on performance by *GlyStruct* is significant compared to *GlyPseAAC*. Significance of contribution and the false discovery rates were also tested for each feature used. All features were found to be significant contributors to the results obtained. The aforementioned test results are included in Additional file 1.

The *GlyNN* webserver [27], which is one of the earliest bioinformatics studies for glycation is still accessible online, however has restrictions of protein sequence length between 34 and 4000 amino acids. Hence, the job we submitted was rejected due to the presence of two protein sequences in our dataset, Q86XX4 of length 4008 amino acids, and P13191 of length 20 amino acids, which violated the *GlyNN* server policies. This webserver was developed using a small dataset curated manually consisting 89 positive and 126 negative glycation sites from 20 peptides, which precedes the recent datasets [30]*.* Moreover, the *GlyNN* authors did not consider residue sites that were not validated at the time of development for training the classifier. These sites marked as “U” to denote “unvalidated site” have since been validated in the recent iteration of the CPLM databank.

Among other recent methods, the webservers for glycation, *PreGly* [40] and *iProtGly-SS* [42] were not functional when accessed to test their method. In addition, the published codes for *Glypre* [41] could not be executed in the absence of a guide. Both *Glypre* and *iProtGly-SS* employed *GlyPse-AAC* data for training their classifier and used *GlyNN* data as comparator dataset. Furthermore, the datasets published by *Glypre* and *iProtGly-SS* were in segmented format without annotating the protein names, therefore could not be used for testing *GlyStruct* predictor. Therefore, pairwise comparison of performance with these state of the art methods was not possible.

With an exception of *GlyNN* and *PreGly,* all other state of the art methods including *GlyStruct* have obtained data from CPLM database. However, there is a significant difference in datasets attributed to regular updates to databanks, the inconsistencies in the selection of primary sequence identity threshold by various authors, and filtering techniques employed to the negative instances of the dataset before training the classifier. Nonetheless, we made comparison with the published results of those methods, which we could not verify through standard means of webservers or codes. The *Glypre* method published high specificity of 0.9078 but recorded average sensitivity of 0.5747 compared to 0.7013 achieved by *GlyStruct*. The accuracy and MCC for *Glypre* were reported to be marginally higher at 0.7968 and 0.52 respectively compared to 0.7562 and 0.51 respectively for *GlyStruct*. Furthermore, *iProtGly-SS* published high sensitivity of 0.9238. However, it recorded lower specificity of 0.6009 compared to 0.7989 by *GlyStruct*. All comparisons are made for 10-fold cross validation which tend to produce best results.

Overall, our predictor *GlyStruct,* using only structural features of peptides and SVM as a classifier produced consistent results (averaged out with 50 runs of cross-validation for each fold) in all the metrics and for all folds. It was better performing than the comparator method, *Gly-PseAAC*. With other state of the art methods on a similar dataset, *GlyStruct* outperformed in one metric or the other by over 10%.

The prime motivation to develop a prediction model for glycation is to for clinical support in timely diagnosis of morbidity and cellular conditions in a cost-effective manner. However, for prediction of PTM like glycation, we need to be mindful of the fact that while sensitivity is highly desired to identify the glycation process, making a false positive prediction can lead to potentially lethal situation. In such cases of false positive prediction, the medical professional may administer medication which would lead to further lowering of blood glucose concentration causing an induced hypoglycemia which can be fatal if not managed well [86, 87]. The prediction model we developed has a low false positive rate (or high specificity) that can be instrumental in avoiding the induced hypoglycemia situation.

## Conclusions

With glycation emerging as one of the clinically important post-translational modification of proteins in recent times, classification engine becomes necessary to predict both, glycated and nonglycated lysine residues with high accuracy. Due to limited dataset and the lack of bias in the sequence motifs attributed to the non-enzymatic nature of this PTM, a great challenge arises to make prediction with high accuracy. The glycation predictor *GlyStruct,* we proposed is based on the secondary structure properties of proteins for which we considered the local backbone angles, secondary structures’ transitional probabilities and the accessible surface area that were obtained through SPIDER2 prediction engine. The protein sequences were truncated into segments of 13 amino acids for each lysine site to produce feature vectors of size (104 × 1). Due to highly unbalanced nature of PTM dataset, *k*-nearest neighbor filtering was employed to balance the classes before training the SVM classifier. The predictor was developed using *libsvm* on WEKA platform and the standard grid-search tuning was applied which yielded better results in comparison to previous studies. The results we obtained has promising levels of robustness due to its relatively high sensitivity of 0.7059 for 8-fold validation, and specificity of over 0.79 in all folds. The latter demonstrates the ability of the predictor to reduce the false positive rate (falsely predicting glycation). For clinical success, higher values for both sensitivity and specificity are desirable for this PTM since false positive prediction can be of more serious concern.

## Additional file

Additional file 1: Significance test for all features and benchmark dataset. (DOCX 44 kb)

## Abbreviations

AGE: Advanced glycation end-products
ASA: Accessible surface area
AUC: Area under the curve
CKSAAP: Composition of 푘-spaced amino acid pairs
CPLM: Compendium of Protein Lysine Modifications
*k*NN: *k*–nearest neighbor
MCC: Mathew’s correlation coefficient
PLMD: Protein Lysine Modification Database
PSAAP: Position-specific amino acid propensity
PTM: Post Translational Modification
RBF: Radial basis function
SVM: Support Vector Machine
