# Supplementary material for: GlyStruct: glycation prediction using structural properties of amino acid residues
Source: BMC Bioinformatics. 2019 Feb 4;19(Suppl 13):547. doi: 10.1186/s12859-018-2547-x (PMC7394324; doi:10.1186/s12859-018-2547-x)
Supplement: Supplementary file 1 — Significance test for all features and benchmark dataset. (DOCX 44 kb) [file 12859_2018_2547_MOESM1_ESM.docx]

Additional file 1

p-values for features included in training

The significant features are highlighted (for p-values < 0.05)

| Feature No. | Feature | p-value | fdr |  | Feature No. | Feature | p-value | fdr |
| --- | --- | --- | --- | --- | --- | --- | --- | --- |
| 1 | ASA -6 | 7.60E-05 | 1.03E-05 |  | 53 | Tau -6 | 5.66E-05 | 7.79E-06 |
| 2 | ASA -5 | 0.224056 | 0.023724 |  | 54 | Tau -5 | 7.15E-09 | 1.12E-09 |
| 3 | ASA -4 | 0.076061 | 0.008869 |  | 55 | Tau -4 | 1.20E-06 | 1.71E-07 |
| 4 | ASA -3 | 1.18E-11 | 2.13E-12 |  | 56 | Tau -3 | 0.001296 | 0.000161 |
| 5 | ASA -2 | 0.119364 | 0.013575 |  | 57 | Tau -2 | 0.000484 | 6.28E-05 |
| 6 | ASA -1 | 1.59E-08 | 2.43E-09 |  | 58 | Tau -1 | 0.000371 | 4.88E-05 |
| 7 | ASA0 | 8.72E-14 | 1.82E-14 |  | 59 | Tau0 | 1.57E-05 | 2.20E-06 |
| 8 | ASA1 | 0.093619 | 0.01078 |  | 60 | Tau1 | 4.44E-12 | 8.70E-13 |
| 9 | ASA2 | 9.06E-11 | 1.52E-11 |  | 61 | Tau2 | 3.56E-08 | 5.37E-09 |
| 10 | ASA3 | 2.29E-11 | 3.98E-12 |  | 62 | Tau3 | 1.54E-07 | 2.29E-08 |
| 11 | ASA4 | 0.619857 | 0.060745 |  | 63 | Tau4 | 1.11E-11 | 2.04E-12 |
| 12 | ASA5 | 0.005014 | 0.000616 |  | 64 | Tau5 | 1.95E-07 | 2.85E-08 |
| 13 | ASA6 | 0.000216 | 2.89E-05 |  | 65 | Tau6 | 6.37E-09 | 1.01E-09 |
| 14 | Psi -6 | 8.85E-19 | 3.02E-19 |  | 66 | SS -Coil -6 | 8.87E-16 | 2.15E-16 |
| 15 | Psi -5 | 9.39E-12 | 1.80E-12 |  | 67 | SS -Coil -5 | 6.45E-19 | 2.28E-19 |
| 16 | Psi -4 | 1.01E-11 | 1.89E-12 |  | 68 | SS -Coil -4 | 8.84E-26 | 2.04E-25 |
| 17 | Psi -3 | 1.28E-11 | 2.28E-12 |  | 69 | SS -Coil -3 | 3.33E-31 | 3.07E-30 |
| 18 | Psi -2 | 8.98E-11 | 1.53E-11 |  | 70 | SS -Coil -2 | 4.02E-26 | 1.24E-25 |
| 19 | Psi -1 | 4.41E-13 | 9.02E-14 |  | 71 | SS -Coil -1 | 1.11E-22 | 1.46E-22 |
| 20 | Psi -0 | 3.24E-20 | 1.49E-20 |  | 72 | SS -Coil0 | 2.22E-22 | 2.28E-22 |
| 21 | Psi1 | 2.07E-25 | 3.81E-25 |  | 73 | SS -Coil1 | 1.61E-19 | 6.74E-20 |
| 22 | Psi2 | 5.66E-18 | 1.63E-18 |  | 74 | SS -Coil2 | 2.31E-15 | 5.45E-16 |
| 23 | Psi3 | 3.10E-19 | 1.24E-19 |  | 75 | SS -Coil3 | 2.15E-20 | 1.17E-20 |
| 24 | Psi4 | 7.53E-18 | 2.04E-18 |  | 76 | SS -Coil4 | 9.20E-21 | 5.30E-21 |
| 25 | Psi5 | 8.85E-19 | 2.91E-19 |  | 77 | SS -Coil5 | 5.06E-21 | 3.33E-21 |
| 26 | Psi6 | 8.89E-19 | 2.82E-19 |  | 78 | SS -Coil6 | 1.21E-21 | 9.26E-22 |
| 27 | Phi -6 | 0.16349 | 0.018367 |  | 79 | SS -Strand -6 | 0.206062 | 0.022072 |
| 28 | Phi -5 | 0.033108 | 0.00391 |  | 80 | SS -Strand -5 | 0.932364 | 0.083387 |
| 29 | Phi -4 | 0.744354 | 0.069262 |  | 81 | SS -Strand -4 | 0.189963 | 0.020832 |
| 30 | Phi -3 | 0.949436 | 0.084097 |  | 82 | SS -Strand -3 | 0.000851 | 0.000107 |
| 31 | Phi -2 | 0.000774 | 9.91E-05 |  | 83 | SS -Strand -2 | 0.008326 | 0.000996 |
| 32 | Phi -1 | 0.385274 | 0.039435 |  | 84 | SS -Strand -1 | 0.192906 | 0.020906 |
| 33 | Phi0 | 0.441786 | 0.044722 |  | 85 | SS -Strand0 | 0.189197 | 0.020998 |
| 34 | Phi1 | 0.655396 | 0.063552 |  | 86 | SS -Strand1 | 0.874845 | 0.07901 |
| 35 | Phi2 | 0.53948 | 0.053437 |  | 87 | SS -Strand2 | 0.280728 | 0.029387 |
| 36 | Phi3 | 0.702987 | 0.06608 |  | 88 | SS -Strand3 | 0.818571 | 0.07466 |
| 37 | Phi4 | 0.005699 | 0.000691 |  | 89 | SS -Strand4 | 0.798439 | 0.073552 |
| 38 | Phi5 | 0.673773 | 0.064654 |  | 90 | SS -Strand5 | 0.528004 | 0.052869 |
| 39 | Phi6 | 0.692806 | 0.065795 |  | 91 | SS -Strand6 | 0.291795 | 0.030202 |
| 40 | Theta -6 | 4.02E-15 | 9.25E-16 |  | 92 | SS -Helix -6 | 5.77E-21 | 3.55E-21 |
| 41 | Theta -5 | 7.97E-16 | 1.99E-16 |  | 93 | SS -Helix -5 | 2.44E-20 | 1.25E-20 |
| 42 | Theta -4 | 4.85E-15 | 1.09E-15 |  | 94 | SS -Helix -4 | 2.43E-21 | 1.72E-21 |
| 43 | Theta -3 | 1.13E-06 | 1.63E-07 |  | 95 | SS -Helix -3 | 4.81E-20 | 2.11E-20 |
| 44 | Theta -2 | 1.12E-10 | 1.85E-11 |  | 96 | SS -Helix -2 | 4.16E-18 | 1.24E-18 |
| 45 | Theta -1 | 6.21E-10 | 1.00E-10 |  | 97 | SS -Helix -1 | 6.11E-18 | 1.70E-18 |
| 46 | Theta0 | 3.62E-14 | 7.75E-15 |  | 98 | SS -Helix0 | 1.09E-17 | 2.88E-18 |
| 47 | Theta1 | 2.44E-18 | 7.49E-19 |  | 99 | SS -Helix1 | 3.58E-19 | 1.37E-19 |
| 48 | Theta2 | 1.58E-14 | 3.46E-15 |  | 100 | SS -Helix2 | 5.71E-19 | 2.10E-19 |
| 49 | Theta3 | 4.44E-12 | 8.89E-13 |  | 101 | SS -Helix3 | 8.01E-22 | 7.38E-22 |
| 50 | Theta4 | 2.76E-20 | 1.34E-20 |  | 102 | SS -Helix4 | 8.24E-22 | 6.90E-22 |
| 51 | Theta5 | 1.14E-22 | 1.32E-22 |  | 103 | SS -Helix5 | 1.41E-24 | 2.17E-24 |
| 52 | Theta6 | 5.34E-16 | 1.37E-16 |  | 104 | SS -Helix6 | 2.12E-27 | 9.77E-27 |

# Dataset

Peptides used in this study (FASTA format)

**>P00325**

**MSTAGKVIKCKAAVLWEVKKPFSIEDVEVAPPKAYEVRIKMVAVGICRTDDHVVSGNLVTPLPVILGHEAAGIVESVGEGVTTVKPGDKVIPLFTPQCGKCRVCKNPESNYCLKNDLGNPRGTLQDGTRRFTCRGKPIHHFLGTSTFSQYTVVDENAVAKIDAASPLEKVCLIGCGFSTGYGSAVNVAKVTPGSTCAVFGLGGVGLSAVMGCKAAGAARIIAVDINKDKFAKAKELGATECINPQDYKKPIQEVLKEMTDGGVDFSFEVIGRLDTMMASLLCCHEACGTSVIVGVPPASQNLSINPMLLLTGRTWKGAVYGGFKSKEGIPKLVADFMAKKFSLDALITHVLPFEKINEGFDLLHSGKSIRTVLTF**

**>P00435**

**MCAAQRSAAALAAAAPRTVYAFSARPLAGGEPFNLSSLRGKVLLIENVASLUGTTVRDYTQMNDLQRRLGPRGLVVLGFPCNQFGHQENAKNEEILNCLKYVRPGGGFEPNFMLFEKCEVNGEKAHPLFAFLREVLPTPSDDATALMTDPKFITWSPVCRNDVSWNFEKFLVGPDGVPVRRYSRRFLTIDIEPDIETLLSQGASA**

**>P00441**

**MATKAVCVLKGDGPVQGIINFEQKESNGPVKVWGSIKGLTEGLHGFHVHEFGDNTAGCTSAGPHFNPLSRKHGGPKDEERHVGDLGNVTADKDGVADVSIEDSVISLSGDHCIIGRTLVVHEKADDLGKGGNEESTKTGNAGSRLACGVIGIAQ**

**>P00450**

**MKILILGIFLFLCSTPAWAKEKHYYIGIIETTWDYASDHGEKKLISVDTEHSNIYLQNGPDRIGRLYKKALYLQYTDETFRTTIEKPVWLGFLGPIIKAETGDKVYVHLKNLASRPYTFHSHGITYYKEHEGAIYPDNTTDFQRADDKVYPGEQYTYMLLATEEQSPGEGDGNCVTRIYHSHIDAPKDIASGLIGPLIICKKDSLDKEKEKHIDREFVVMFSVVDENFSWYLEDNIKTYCSEPEKVDKDNEDFQESNRMYSVNGYTFGSLPGLSMCAEDRVKWYLFGMGNEVDVHAAFFHGQALTNKNYRIDTINLFPATLFDAYMVAQNPGEWMLSCQNLNHLKAGLQAFFQVQECNKSSSKDNIRGKHVRHYYIAAEEIIWNYAPSGIDIFTKENLTAPGSDSAVFFEQGTTRIGGSYKKLVYREYTDASFTNRKERGPEEEHLGILGPVIWAEVGDTIRVTFHNKGAYPLSIEPIGVRFNKNNEGTYYSPNYNPQSRSVPPSASHVAPTETFTYEWTVPKEVGPTNADPVCLAKMYYSAVDPTKDIFTGLIGPMKICKKGSLHANGRQKDVDKEFYLFPTVFDENESLLLEDNIRMFTTAPDQVDKEDEDFQESNKMHSMNGFMYGNQPGLTMCKGDSVVWYLFSAGNEADVHGIYFSGNTYLWRGERRDTANLFPQTSLTLHMWPDTEGTFNVECLTTDHYTGGMKQKYTVNQCRRQSEDSTFYLGERTYYIAAVEVEWDYSPQREWEKELHHLQEQNVSNAFLDKGEFYIGSKYKKVVYRQYTDSTFRVPVERKAEEEHLGILGPQLHADVGDKVKIIFKNMATRPYSIHAHGVQTESSTVTPTLPGETLTYVWKIPERSGAGTEDSACIPWAYYSTVDQVKDLYSGLIGPLIVCRRPYLKVFNPRRKLEFALLFLVFDENESWYLDDNIKTYSDHPEKVNKDDEEFIESNKMHAINGRMFGNLQGLTMHVGDEVNWYLMGMGNEIDLHTVHFHGHSFQYKHRGVYSSDVFDIFPGTYQTLEMFPRTPGIWLLHCHVTDHIHAGMETTYTVLQNEDTKSG**

**>P00698**

**MRSLLILVLCFLPLAALGKVFGRCELAAAMKRHGLDNYRGYSLGNWVCAAKFESNFNTQATNRNTDGSTDYGILQINSRWWCNDGRTPGSRNLCNIPCSALLSSDITASVNCAKKIVSDGNGMNAWVAWRNRCKGTDVQAWIRGCRL**

**>P00738**

**MSALGAVIALLLWGQLFAVDSGNDVTDIADDGCPKPPEIAHGYVEHSVRYQCKNYYKLRTEGDGVYTLNDKKQWINKAVGDKLPECEADDGCPKPPEIAHGYVEHSVRYQCKNYYKLRTEGDGVYTLNNEKQWINKAVGDKLPECEAVCGKPKNPANPVQRILGGHLDAKGSFPWQAKMVSHHNLTTGATLINEQWLLTTAKNLFLNHSENATAKDIAPTLTLYVGKKQLVEIEKVVLHPNYSQVDIGLIKLKQKVSVNERVMPICLPSKDYAEVGRVGYVSGWGRNANFKFTDHLKYVMLPVADQDQCIRHYEGSTVPEKKTPKSPVGVQPILNEHTFCAGMSKYQEDTCYGDAGSAFAVHDLEEDTWYATGILSFDKSCAVAEYGVYVKVTSIQDWVQKTIAEN**

**>P00751**

**MGSNLSPQLCLMPFILGLLSGGVTTTPWSLARPQGSCSLEGVEIKGGSFRLLQEGQALEYVCPSGFYPYPVQTRTCRSTGSWSTLKTQDQKTVRKAECRAIHCPRPHDFENGEYWPRSPYYNVSDEISFHCYDGYTLRGSANRTCQVNGRWSGQTAICDNGAGYCSNPGIPIGTRKVGSQYRLEDSVTYHCSRGLTLRGSQRRTCQEGGSWSGTEPSCQDSFMYDTPQEVAEAFLSSLTETIEGVDAEDGHGPGEQQKRKIVLDPSGSMNIYLVLDGSDSIGASNFTGAKKCLVNLIEKVASYGVKPRYGLVTYATYPKIWVKVSEADSSNADWVTKQLNEINYEDHKLKSGTNTKKALQAVYSMMSWPDDVPPEGWNRTRHVIILMTDGLHNMGGDPITVIDEIRDLLYIGKDRKNPREDYLDVYVFGVGPLVNQVNINALASKKDNEQHVFKVKDMENLEDVFYQMIDESQSLSLCGMVWEHRKGTDYHKQPWQAKISVIRPSKGHESCMGAVVSEYFVLTAAHCFTVDDKEHSIKVSVGGEKRDLEIEVVLFHPNYNINGKKEAGIPEFYDYDVALIKLKNKLKYGQTIRPICLPCTEGTTRALRLPPTTTCQQQKEELLPAQDIKALFVSEEEKKLTRKEVYIKNGDKKGSCERDAQYAPGYDKVKDISEVVTPRFLCTGGVSPYADPNTCRGDSGGPLIVHKRSRFIQVGVISWGVVDVCKNQKRQKQVPAHARDFHINLFQVLPWLKEKLQDEDLGFL**

**>P01009**

**MPSSVSWGILLLAGLCCLVPVSLAEDPQGDAAQKTDTSHHDQDHPTFNKITPNLAEFAFSLYRQLAHQSNSTNIFFSPVSIATAFAMLSLGTKADTHDEILEGLNFNLTEIPEAQIHEGFQELLRTLNQPDSQLQLTTGNGLFLSEGLKLVDKFLEDVKKLYHSEAFTVNFGDTEEAKKQINDYVEKGTQGKIVDLVKELDRDTVFALVNYIFFKGKWERPFEVKDTEEEDFHVDQVTTVKVPMMKRLGMFNIQHCKKLSSWVLLMKYLGNATAIFFLPDEGKLQHLENELTHDIITKFLENEDRRSASLHLPKLSITGTYDLKSVLGQLGITKVFSNGADLSGVTEEAPLKLSKAVHKAVLTIDEKGTEAAGAMFLEAIPMSIPPEVKFNKPFVFLMIEQNTKSPLFMGKVVNPTQK**

**>P01317**

**MALWTRLRPLLALLALWPPPPARAFVNQHLCGSHLVEALYLVCGERGFFYTPKARREVEGPQVGALELAGGPGAGGLEGPPQKRGIVEQCCASVCSLYQLENYCN**

**>P01834**

**TVAAPSVFIFPPSDEQLKSGTASVVCLLNNFYPREAKVQWKVDNALQSGNSQESVTEQDSKDSTYSLSSTLTLSKADYEKHKVYACEVTHQGLSSPVTKSFNRGEC**

**>P01876**

**ASPTSPKVFPLSLCSTQPDGNVVIACLVQGFFPQEPLSVTWSESGQGVTARNFPPSQDASGDLYTTSSQLTLPATQCLAGKSVTCHVKHYTNPSQDVTVPCPVPSTPPTPSPSTPPTPSPSCCHPRLSLHRPALEDLLLGSEANLTCTLTGLRDASGVTFTWTPSSGKSAVQGPPERDLCGCYSVSSVLPGCAEPWNHGKTFTCTAAYPESKTPLTATLSKSGNTFRPEVHLLPPPSEELALNELVTLTCLARGFSPKDVLVRWLQGSQELPREKYLTWASRQEPSQGTTTFAVTSILRVAAEDWKKGDTFSCMVGHEALPLAFTQKTIDRLAGKPTHVNVSVVMAEVDGTCY**

**>P02453**

**MFSFVDLRLLLLLAATALLTHGQEEGQEEGQEEDIPPVTCVQNGLRYHDRDVWKPVPCQICVCDNGNVLCDDVICDELKDCPNAKVPTDECCPVCPEGQESPTDQETTGVEGPKGDTGPRGPRGPAGPPGRDGIPGQPGLPGPPGPPGPPGPPGLGGNFAPQLSYGYDEKSTGISVPGPMGPSGPRGLPGPPGAPGPQGFQGPPGEPGEPGASGPMGPRGPPGPPGKNGDDGEAGKPGRPGERGPPGPQGARGLPGTAGLPGMKGHRGFSGLDGAKGDAGPAGPKGEPGSPGENGAPGQMGPRGLPGERGRPGAPGPAGARGNDGATGAAGPPGPTGPAGPPGFPGAVGAKGEGGPQGPRGSEGPQGVRGEPGPPGPAGAAGPAGNPGADGQPGAKGANGAPGIAGAPGFPGARGPSGPQGPSGPPGPKGNSGEPGAPGSKGDTGAKGEPGPTGIQGPPGPAGEEGKRGARGEPGPAGLPGPPGERGGPGSRGFPGADGVAGPKGPAGERGAPGPAGPKGSPGEAGRPGEAGLPGAKGLTGSPGSPGPDGKTGPPGPAGQDGRPGPPGPPGARGQAGVMGFPGPKGAAGEPGKAGERGVPGPPGAVGPAGKDGEAGAQGPPGPAGPAGERGEQGPAGSPGFQGLPGPAGPPGEAGKPGEQGVPGDLGAPGPSGARGERGFPGERGVQGPPGPAGPRGANGAPGNDGAKGDAGAPGAPGSQGAPGLQGMPGERGAAGLPGPKGDRGDAGPKGADGAPGKDGVRGLTGPIGPPGPAGAPGDKGEAGPSGPAGPTGARGAPGDRGEPGPPGPAGFAGPPGADGQPGAKGEPGDAGAKGDAGPPGPAGPAGPPGPIGNVGAPGPKGARGSAGPPGATGFPGAAGRVGPPGPSGNAGPPGPPGPAGKEGSKGPRGETGPAGRPGEVGPPGPPGPAGEKGAPGADGPAGAPGTPGPQGIAGQRGVVGLPGQRGERGFPGLPGPSGEPGKQGPSGASGERGPPGPMGPPGLAGPPGESGREGAPGAEGSPGRDGSPGAKGDRGETGPAGPPGAPGAPGAPGPVGPAGKSGDRGETGPAGPAGPIGPVGARGPAGPQGPRGDKGETGEQGDRGIKGHRGFSGLQGPPGPPGSPGEQGPSGASGPAGPRGPPGSAGSPGKDGLNGLPGPIGPPGPRGRTGDAGPAGPPGPPGPPGPPGPPSGGYDLSFLPQPPQEKAHDGGRYYRADDANVVRDRDLEVDTTLKSLSQQIENIRSPEGSRKNPARTCRDLKMCHSDWKSGEYWIDPNQGCNLDAIKVFCNMETGETCVYPTQPSVAQKNWYISKNPKEKRHVWYGESMTGGFQFEYGGQGSDPADVAIQLTFLRLMSTEASQNITYHCKNSVAYMDQQTGNLKKALLLQGSNEIEIRAEGNSRFTYSVTYDGCTSHTGAWGKTVIEYKTTKTSRLPIIDVAPLDVGAPDQEFGFDVGPACFL**

**>P02470**

**MDIAIQHPWFKRTLGPFYPSRLFDQFFGEGLFEYDLLPFLSSTISPYYRQSLFRTVLDSGISEVRSDRDKFVIFLDVKHFSPEDLTVKVQEDFVEIHGKHNERQDDHGYISREFHRRYRLPSNVDQSALSCSLSADGMLTFSGPKIPSGVDAGHSERAIPVSREEKPSSAPSS**

**>P02522**

**MASDHQTQAGKPQPLNPKIIIFEQENFQGHSHELNGPCPNLKETGVEKAGSVLVQAGPWVGYEQANCKGEQFVFEKGEYPRWDSWTSSRRTDSLSSLRPIKVDSQEHKITLYENPNFTGKKMEVIDDDVPSFHAHGYQEKVSSVRVQSGTWVGYQYPGYRGLQYLLEKGDYKDSGDFGAPQPQVQSVRRIRDMQWHQRGAFHPSS**

**>P02526**

**MGKITFYEDRGFQGHCYECSSDCPNLQPYFSRCNSIRVDSGCWMLYERPNYQGHQYFLRRGDYPDYQQWMGFNDSIRSCRLIPQHTGTFRMRIYERDDFRGQMSEITDDCPSLQDRFHLTEVHSLNVLEGSWVLYEMPSYRGRQYLLRPGEYRRYLDWGAMNAKVGSLRRVMDFY**

**>P02647**

**MKAAVLTLAVLFLTGSQARHFWQQDEPPQSPWDRVKDLATVYVDVLKDSGRDYVSQFEGSALGKQLNLKLLDNWDSVTSTFSKLREQLGPVTQEFWDNLEKETEGLRQEMSKDLEEVKAKVQPYLDDFQKKWQEEMELYRQKVEPLRAELQEGARQKLHELQEKLSPLGEEMRDRARAHVDALRTHLAPYSDELRQRLAARLEALKENGGARLAEYHAKATEHLSTLSEKAKPALEDLRQGLLPVLESFKVSFLSALEEYTKKLNTQ**

**>P02649**

**MKVLWAALLVTFLAGCQAKVEQAVETEPEPELRQQTEWQSGQRWELALGRFWDYLRWVQTLSEQVQEELLSSQVTQELRALMDETMKELKAYKSELEEQLTPVAEETRARLSKELQAAQARLGADMEDVCGRLVQYRGEVQAMLGQSTEELRVRLASHLRKLRKRLLRDADDLQKRLAVYQAGAREGAERGLSAIRERLGPLVEQGRVRAATVGSLAGQPLQERAQAWGERLRARMEEMGSRTRDRLDEVKEQVAEVRAKLEEQAQQIRLQAEAFQARLKSWFEPLVEDMQRQWAGLVEKVQAAVGTSAAPVPSDNH**

**>P02652**

**MKLLAATVLLLTICSLEGALVRRQAKEPCVESLVSQYFQTVTDYGKDLMEKVKSPELQAEAKSYFEKSKEQLTPLIKKAGTELVNFLSYFVELGTQPATQ**

**>P02671**

**MFSMRIVCLVLSVVGTAWTADSGEGDFLAEGGGVRGPRVVERHQSACKDSDWPFCSDEDWNYKCPSGCRMKGLIDEVNQDFTNRINKLKNSLFEYQKNNKDSHSLTTNIMEILRGDFSSANNRDNTYNRVSEDLRSRIEVLKRKVIEKVQHIQLLQKNVRAQLVDMKRLEVDIDIKIRSCRGSCSRALAREVDLKDYEDQQKQLEQVIAKDLLPSRDRQHLPLIKMKPVPDLVPGNFKSQLQKVPPEWKALTDMPQMRMELERPGGNEITRGGSTSYGTGSETESPRNPSSAGSWNSGSSGPGSTGNRNPGSSGTGGTATWKPGSSGPGSTGSWNSGSSGTGSTGNQNPGSPRPGSTGTWNPGSSERGSAGHWTSESSVSGSTGQWHSESGSFRPDSPGSGNARPNNPDWGTFEEVSGNVSPGTRREYHTEKLVTSKGDKELRTGKEKVTSGSTTTTRRSCSKTVTKTVIGPDGHKEVTKEVVTSEDGSDCPEAMDLGTLSGIGTLDGFRHRHPDEAAFFDTASTGKTFPGFFSPMLGEFVSETESRGSESGIFTNTKESSSHHPGIAEFPSRGKSSSYSKQFTSSTSYNRGDSTFESKSYKMADEAGSEADHEGTHSTKRGHAKSRPVRDCDDVLQTHPSGTQSGIFNIKLPGSSKIFSVYCDQETSLGGWLLIQQRMDGSLNFNRTWQDYKRGFGSLNDEGEGEFWLGNDYLHLLTQRGSVLRVELEDWAGNEAYAEYHFRVGSEAEGYALQVSSYEGTAGDALIEGSVEEGAEYTSHNNMQFSTFDRDADQWEENCAEVYGGGWWYNNCQAANLNGIYYPGGSYDPRNNSPYEIENGVVWVSFRGADYSLRAVRMKIRPLVTQ**

**>P02675**

**MKRMVSWSFHKLKTMKHLLLLLLCVFLVKSQGVNDNEEGFFSARGHRPLDKKREEAPSLRPAPPPISGGGYRARPAKAAATQKKVERKAPDAGGCLHADPDLGVLCPTGCQLQEALLQQERPIRNSVDELNNNVEAVSQTSSSSFQYMYLLKDLWQKRQKQVKDNENVVNEYSSELEKHQLYIDETVNSNIPTNLRVLRSILENLRSKIQKLESDVSAQMEYCRTPCTVSCNIPVVSGKECEEIIRKGGETSEMYLIQPDSSVKPYRVYCDMNTENGGWTVIQNRQDGSVDFGRKWDPYKQGFGNVATNTDGKNYCGLPGEYWLGNDKISQLTRMGPTELLIEMEDWKGDKVKAHYGGFTVQNEANKYQISVNKYRGTAGNALMDGASQLMGENRTMTIHNGMFFSTYDRDNDGWLTSDPRKQCSKEDGGGWWYNRCHAANPNGRYYWGGQYTWDMAKHGTDDGVVWMNWKGSWYSMRKMSMKIRPFFPQQ**

**>P02679**

**MSWSLHPRNLILYFYALLFLSSTCVAYVATRDNCCILDERFGSYCPTTCGIADFLSTYQTKVDKDLQSLEDILHQVENKTSEVKQLIKAIQLTYNPDESSKPNMIDAATLKSRKMLEEIMKYEASILTHDSSIRYLQEIYNSNNQKIVNLKEKVAQLEAQCQEPCKDTVQIHDITGKDCQDIANKGAKQSGLYFIKPLKANQQFLVYCEIDGSGNGWTVFQKRLDGSVDFKKNWIQYKEGFGHLSPTGTTEFWLGNEKIHLISTQSAIPYALRVELEDWNGRTSTADYAMFKVGPEADKYRLTYAYFAGGDAGDAFDGFDFGDDPSDKFFTSHNGMQFSTWDNDNDKFEGNCAEQDGSGWWMNKCHAGHLNGVYYQGGTYSKASTPNGYDNGIIWATWKTRWYSMKKTTMKIIPFNRLTIGEGQQHHLGGAKQVRPEHPAETEYDSLYPEDDL**

**>P02763**

**MALSWVLTVLSLLPLLEAQIPLCANLVPVPITNATLDQITGKWFYIASAFRNEEYNKSVQEIQATFFYFTPNKTEDTIFLREYQTRQDQCIYNTTYLNVQRENGTISRYVGGQEHFAHLLILRDTKTYMLAFDVNDEKNWGLSVYADKPETTKEQLGEFYEALDCLRIPKSDVVYTDWKKDKCEPLEKQHEKERKQEEGES**

**>P02768**

**MKWVTFISLLFLFSSAYSRGVFRRDAHKSEVAHRFKDLGEENFKALVLIAFAQYLQQCPFEDHVKLVNEVTEFAKTCVADESAENCDKSLHTLFGDKLCTVATLRETYGEMADCCAKQEPERNECFLQHKDDNPNLPRLVRPEVDVMCTAFHDNEETFLKKYLYEIARRHPYFYAPELLFFAKRYKAAFTECCQAADKAACLLPKLDELRDEGKASSAKQRLKCASLQKFGERAFKAWAVARLSQRFPKAEFAEVSKLVTDLTKVHTECCHGDLLECADDRADLAKYICENQDSISSKLKECCEKPLLEKSHCIAEVENDEMPADLPSLAADFVESKDVCKNYAEAKDVFLGMFLYEYARRHPDYSVVLLLRLAKTYETTLEKCCAAADPHECYAKVFDEFKPLVEEPQNLIKQNCELFEQLGEYKFQNALLVRYTKKVPQVSTPTLVEVSRNLGKVGSKCCKHPEAKRMPCAEDYLSVVLNQLCVLHEKTPVSDRVTKCCTESLVNRRPCFSALEVDETYVPKEFNAETFTFHADICTLSEKERQIKKQTALVELVKHKPKATKEQLKAVMDDFAAFVEKCCKADDKETCFAEEGKKLVAASQAALGL**

**>P02774**

**MKRVLVLLLAVAFGHALERGRDYEKNKVCKEFSHLGKEDFTSLSLVLYSRKFPSGTFEQVSQLVKEVVSLTEACCAEGADPDCYDTRTSALSAKSCESNSPFPVHPGTAECCTKEGLERKLCMAALKHQPQEFPTYVEPTNDEICEAFRKDPKEYANQFMWEYSTNYGQAPLSLLVSYTKSYLSMVGSCCTSASPTVCFLKERLQLKHLSLLTTLSNRVCSQYAAYGEKKSRLSNLIKLAQKVPTADLEDVLPLAEDITNILSKCCESASEDCMAKELPEHTVKLCDNLSTKNSKFEDCCQEKTAMDVFVCTYFMPAAQLPELPDVELPTNKDVCDPGNTKVMDKYTFELSRRTHLPEVFLSKVLEPTLKSLGECCDVEDSTTCFNAKGPLLKKELSSFIDKGQELCADYSENTFTEYKKKLAERLKAKLPDATPKELAKLVNKRSDFASNCCSINSPPLYCDSEIDAELKNIL**

**>P02787**

**MRLAVGALLVCAVLGLCLAVPDKTVRWCAVSEHEATKCQSFRDHMKSVIPSDGPSVACVKKASYLDCIRAIAANEADAVTLDAGLVYDAYLAPNNLKPVVAEFYGSKEDPQTFYYAVAVVKKDSGFQMNQLRGKKSCHTGLGRSAGWNIPIGLLYCDLPEPRKPLEKAVANFFSGSCAPCADGTDFPQLCQLCPGCGCSTLNQYFGYSGAFKCLKDGAGDVAFVKHSTIFENLANKADRDQYELLCLDNTRKPVDEYKDCHLAQVPSHTVVARSMGGKEDLIWELLNQAQEHFGKDKSKEFQLFSSPHGKDLLFKDSAHGFLKVPPRMDAKMYLGYEYVTAIRNLREGTCPEAPTDECKPVKWCALSHHERLKCDEWSVNSVGKIECVSAETTEDCIAKIMNGEADAMSLDGGFVYIAGKCGLVPVLAENYNKSDNCEDTPEAGYFAIAVVKKSASDLTWDNLKGKKSCHTAVGRTAGWNIPMGLLYNKINHCRFDEFFSEGCAPGSKKDSSLCKLCMGSGLNLCEPNNKEGYYGYTGAFRCLVEKGDVAFVKHQTVPQNTGGKNPDPWAKNLNEKDYELLCLDGTRKPVEEYANCHLARAPNHAVVTRKDKEACVHKILRQQQHLFGSNVTDCSGNFCLFRSETKDLLFRDDTVCLAKLHDRNTYEKYLGEEYVKAVGNLRKCSTSSLLEACTFRRP**

**>P02790**

**MARVLGAPVALGLWSLCWSLAIATPLPPTSAHGNVAEGETKPDPDVTERCSDGWSFDATTLDDNGTMLFFKGEFVWKSHKWDRELISERWKNFPSPVDAAFRQGHNSVFLIKGDKVWVYPPEKKEKGYPKLLQDEFPGIPSPLDAAVECHRGECQAEGVLFFQGDREWFWDLATGTMKERSWPAVGNCSSALRWLGRYYCFQGNQFLRFDPVRGEVPPRYPRDVRDYFMPCPGRGHGHRNGTGHGNSTHHGPEYMRCSPHLVLSALTSDNHGATYAFSGTHYWRLDTSRDGWHSWPIAHQWPQGPSAVDAAFSWEEKLYLVQGTQVYVFLTKGGYTLVSGYPKRLEKEVGTPHGIILDSVDAAFICPGSSRLHIMAGRRLWWLDLKSGAQATWTELPWPHEKVDGALCMEKSLGPNSCSANGPGLYLIHGPNLYCYSDVEKLNAAKALPQPQNVTSLLGCTH**

**>P04792**

**MTERRVPFSLLRGPSWDPFRDWYPHSRLFDQAFGLPRLPEEWSQWLGGSSWPGYVRPLPPAAIESPAVAAPAYSRALSRQLSSGVSEIRHTADRWRVSLDVNHFAPDELTVKTKDGVVEITGKHEERQDEHGYISRCFTRKYTLPPGVDPTQVSSSLSPEGTLTVEAPMPKLATQSNEITIPVTFESRAQLGGPEAAKSDETAAK**

**>P06624**

**MWELRSASFWRAICAEFFASLFYVFFGLGASLRWAPGPLHVLQVALAFGLALATLVQAVGHISGAHVNPAVTFAFLVGSQMSLLRAICYMVAQLLGAVAGAAVLYSVTPPAVRGNLALNTLHPGVSVGQATIVEIFLTLQFVLCIFATYDERRNGRLGSVALAVGFSLTLGHLFGMYYTGAGMNPARSFAPAILTRNFTNHWVYWVGPVIGAGLGSLLYDFLLFPRLKSVSERLSILKGSRPSESNGQPEVTGEPVELKTQAL**

**>P07435**

**AQEEEAEQNLSELSGPWRTVYIGSTNPEKIQENGPFRTYFRELVFDDEKGTVDFYFSVKRDGKWKNVHVKATKQDDGTYVADYEGQNVFKIVSLSRTHLVAHNINVDKHGQTTELTELFVKLNVEDEDLEKFWKLTEDKGIDKKNVVNFLENEDHPHPE**

**>P07738**

**MSKYKLIMLRHGEGAWNKENRFCSWVDQKLNSEGMEEARNCGKQLKALNFEFDLVFTSVLNRSIHTAWLILEELGQEWVPVESSWRLNERHYGALIGLNREQMALNHGEEQVRLWRRSYNVTPPPIEESHPYYQEIYNDRRYKVCDVPLDQLPRSESLKDVLERLLPYWNERIAPEVLRGKTILISAHGNSSRALLKHLEGISDEDIINITLPTGVPILLELDENLRAVGPHQFLGDQEAIQAAIKKVEDQGKVKQAKK**

**>P08294**

**MLALLCSCLLLAAGASDAWTGEDSAEPNSDSAEWIRDMYAKVTEIWQEVMQRRDDDGALHAACQVQPSATLDAAQPRVTGVVLFRQLAPRAKLDAFFALEGFPTEPNSSSRAIHVHQFGDLSQGCESTGPHYNPLAVPHPQHPGDFGNFAVRDGSLWRYRAGLAASLAGPHSIVGRAVVVHAGEDDLGRGGNQASVENGNAGRRLACCVVGVCGPGLWERQAREHSERKKRRRESECKAA**

**>P08670**

**MSTRSVSSSSYRRMFGGPGTASRPSSSRSYVTTSTRTYSLGSALRPSTSRSLYASSPGGVYATRSSAVRLRSSVPGVRLLQDSVDFSLADAINTEFKNTRTNEKVELQELNDRFANYIDKVRFLEQQNKILLAELEQLKGQGKSRLGDLYEEEMRELRRQVDQLTNDKARVEVERDNLAEDIMRLREKLQEEMLQREEAENTLQSFRQDVDNASLARLDLERKVESLQEEIAFLKKLHEEEIQELQAQIQEQHVQIDVDVSKPDLTAALRDVRQQYESVAAKNLQEAEEWYKSKFADLSEAANRNNDALRQAKQESTEYRRQVQSLTCEVDALKGTNESLERQMREMEENFAVEAANYQDTIGRLQDEIQNMKEEMARHLREYQDLLNVKMALDIEIATYRKLLEGEESRISLPLPNFSSLNLRETNLDSLPLVDTHSKRTLLIKTVETRDGQVINETSQHHDDLE**

**>P10591**

**MSKAVGIDLGTTYSCVAHFANDRVDIIANDQGNRTTPSFVAFTDTERLIGDAAKNQAAMNPSNTVFDAKRLIGRNFNDPEVQADMKHFPFKLIDVDGKPQIQVEFKGETKNFTPEQISSMVLGKMKETAESYLGAKVNDAVVTVPAYFNDSQRQATKDAGTIAGLNVLRIINEPTAAAIAYGLDKKGKEEHVLIFDLGGGTFDVSLLSIEDGIFEVKATAGDTHLGGEDFDNRLVNHFIQEFKRKNKKDLSTNQRALRRLRTACERAKRTLSSSAQTSVEIDSLFEGIDFYTSITRARFEELCADLFRSTLDPVEKVLRDAKLDKSQVDEIVLVGGSTRIPKVQKLVTDYFNGKEPNRSINPDEAVAYGAAVQAAILTGDESSKTQDLLLLDVAPLSLGIETAGGVMTKLIPRNSTIPTKKSEIFSTYADNQPGVLIQVFEGERAKTKDNNLLGKFELSGIPPAPRGVPQIEVTFDVDSNGILNVSAVEKGTGKSNKITITNDKGRLSKEDIEKMVAEAEKFKEEDEKESQRIASKNQLESIAYSLKNTISEAGDKLEQADKDTVTKKAEETISWLDSNTTASKEEFDDKLKELQDIANPIMSKLYQAGGAPGGAAGGAPGGFPGGAPPAPEAEGPTVEEVD**

**>P10636**

**MAEPRQEFEVMEDHAGTYGLGDRKDQGGYTMHQDQEGDTDAGLKESPLQTPTEDGSEEPGSETSDAKSTPTAEDVTAPLVDEGAPGKQAAAQPHTEIPEGTTAEEAGIGDTPSLEDEAAGHVTQEPESGKVVQEGFLREPGPPGLSHQLMSGMPGAPLLPEGPREATRQPSGTGPEDTEGGRHAPELLKHQLLGDLHQEGPPLKGAGGKERPGSKEEVDEDRDVDESSPQDSPPSKASPAQDGRPPQTAAREATSIPGFPAEGAIPLPVDFLSKVSTEIPASEPDGPSVGRAKGQDAPLEFTFHVEITPNVQKEQAHSEEHLGRAAFPGAPGEGPEARGPSLGEDTKEADLPEPSEKQPAAAPRGKPVSRVPQLKARMVSKSKDGTGSDDKKAKTSTRSSAKTLKNRPCLSPKHPTPGSSDPLIQPSSPAVCPEPPSSPKYVSSVTSRTGSSGAKEMKLKGADGKTKIATPRGAAPPGQKGQANATRIPAKTPPAPKTPPSSGEPPKSGDRSGYSSPGSPGTPGSRSRTPSLPTPPTREPKKVAVVRTPPKSPSSAKSRLQTAPVPMPDLKNVKSKIGSTENLKHQPGGGKVQIINKKLDLSNVQSKCGSKDNIKHVPGGGSVQIVYKPVDLSKVTSKCGSLGNIHHKPGGGQVEVKSEKLDFKDRVQSKIGSLDNITHVPGGGNKKIETHKLTFRENAKAKTDHGAEIVYKSPVVSGDTSPRHLSNVSSTGSIDMVDSPQLATLADEVSASLAKQGL**

**>P11277**

**MTSATEFENVGNQPPYSRINARWDAPDDELDNDNSSARLFERSRIKALADEREVVQKKTFTKWVNSHLARVSCRITDLYKDLRDGRMLIKLLEVLSGEMLPKPTKGKMRIHCLENVDKALQFLKEQRVHLENMGSHDIVDGNHRLVLGLIWTIILRFQIQDIVVQTQEGRETRSAKDALLLWCQMKTAGYPHVNVTNFTSSWKDGLAFNALIHKHRPDLIDFDKLKDSNARHNLEHAFNVAERQLGIIPLLDPEDVFTENPDEKSIITYVVAFYHYFSKMKVLAVEGKRVGKVIDHAIETEKMIEKYSGLASDLLTWIEQTITVLNSRKFANSLTGVQQQLQAFSTYRTVEKPPKFQEKGNLEVLLFTIQSRMRANNQKVYTPHDGKLVSDINRAWESLEEAEYRRELALRNELIRQEKLEQLARRFDRKAAMRETWLSENQRLVAQDNFGYDLAAVEAAKKKHEAIETDTAAYEERVRALEDLAQELEKENYHDQKRITARKDNILRLWSYLQELLQSRRQRLETTLALQKLFQDMLHSIDWMDEIKAHLLSAEFGKHLLEVEDLLQKHKLMEADIAIQGDKVKAITAATLKFTEGKGYQPCDPQVIQDRISHLEQCFEELSNMAAGRKAQLEQSKRLWKFFWEMDEAESWIKEKEQIYSSLDYGKDLTSVLILQRKHKAFEDELRGLDAHLEQIFQEAHGMVARKQFGHPQIEARIKEVSAQWDQLKDLAAFCKKNLQDAENFFQFQGDADDLKAWLQDAHRLLSGEDVGQDEGATRALGKKHKDFLEELEESRGVMEHLEQQAQGFPEEFRDSPDVTHRLQALRELYQQVVAQADLRQQRLQEALDLYTVFGETDACELWMGEKEKWLAEMEMPDTLEDLEVVQHRFDILDQEMKTLMTQIDGVNLAANSLVESGHPRSREVKQYQDHLNTRWQAFQTLVSERREAVDSALRVHNYCVDCEETSKWITDKTKVVESTKDLGRDLAGIIAIQRKLSGLERDVAAIQARVDALERESQQLMDSHPEQKEDIGQRQKHLEELWQGLQQSLQGQEDLLGEVSQLQAFLQDLDDFQAWLSITQKAVASEDMPESLPEAEQLLQQHAGIKDEIDGHQDSYQRVKESGEKVIQGQTDPEYLLLGQRLEGLDTGWNALGRMWESRSHTLAQCLGFQEFQKDAKQAEAILSNQEYTLAHLEPPDSLEAAEAGIRKFEDFLGSMENNRDKVLSPVDSGNKLVAEGNLYSDKIKEKVQLIEDRHRKNNEKAQEASVLLRDNLELQNFLQNCQELTLWINDKLLTSQDVSYDEARNLHNKWLKHQAFVAELASHEGWLENIDAEGKQLMDEKPQFTALVSQKLEALHRLWDELQATTKEKTQHLSAARSSDLRLQTHADLNKWISAMEDQLRSDDPGKDLTSVNRMLAKLKRVEDQVNVRKEELGELFAQVPSMGEEGGDADLSIEKRFLDLLEPLGRRKKQLESSRAKLQISRDLEDETLWVEERLPLAQSADYGTNLQTVQLFMKKNQTLQNEILGHTPRVEDVLQRGQQLVEAAEIDCQDLEERLGHLQSSWDRLREAAAGRLQRLRDANEAQQYYLDADEAEAWIGEQELYVISDEIPKDEEGAIVMLKRHLRQQRAVEDYGRNIKQLASRAQGLLSAGHPEGEQIIRLQGQVDKHYAGLKDVAEERKRKLENMYHLFQLKRETDDLEQWISEKELVASSPEMGQDFDHVTLLRDKFRDFARETGAIGQERVDNVNAFIERLIDAGHSEAATIAEWKDGLNEMWADLLELIDTRMQLLAASYDLHRYFYTGAEILGLIDEKHRELPEDVGLDASTAESFHRVHTAFERELHLLGVQVQQFQDVATRLQTAYAGEKAEAIQNKEQEVSAAWQALLDACAGRRTQLVDTADKFRFFSMARDLLSWMESIIRQIETQERPRDVSSVELLMKYHQGINAEIETRSKNFSACLELGESLLQRQHQASEEIREKLQQVMSRRKEMNEKWEARWERLRMLLEVCQFSRDASVAEAWLIAQEPYLASGDFGHTVDSVEKLIKRHEAFEKSTASWAERFAALEKPTTLELKERQIAERPAEETGPQEEEGETAGEAPVSHHAATERTSPVSLWSRLSSSWESLQPEPSHPY**

**>P11413**

**MAEQVALSRTQVCGILREELFQGDAFHQSDTHIFIIMGASGDLAKKKIYPTIWWLFRDGLLPENTFIVGYARSRLTVADIRKQSEPFFKATPEEKLKLEDFFARNSYVAGQYDDAASYQRLNSHMNALHLGSQANRLFYLALPPTVYEAVTKNIHESCMSQIGWNRIIVEKPFGRDLQSSDRLSNHISSLFREDQIYRIDHYLGKEMVQNLMVLRFANRIFGPIWNRDNIACVILTFKEPFGTEGRGGYFDEFGIIRDVMQNHLLQMLCLVAMEKPASTNSDDVRDEKVKVLKCISEVQANNVVLGQYVGNPDGEGEATKGYLDDPTVPRGSTTATFAAVVLYVENERWDGVPFILRCGKALNERKAEVRLQFHDVAGDIFHQQCKRNELVIRVQPNEAVYTKMMTKKPGMFFNPEESELDLTYGNRYKNVKLPDAYERLILDVFCGSQMHFVRSDELREAWRIFTPLLHQIELEKPKPIPYIYGSRGPTEADELMKRVGFQYEGTYKWVNPHKL**

**>P13191**

**QELASFHSVSKGFMGECGFR**

**>P13987**

**MGIQGGSVLFGLLLVLAVFCHSGHSLQCYNCPNPTADCKTAVNCSSDFDACLITKAGLQVYNKCWKFEHCNFNDVTTRLRENELTYYCCKKDLCNFNEQLENGGTSLSEKTVLLLVTPFLAAAWSLHP**

**>P16402**

**MSETAPLAPTIPAPAEKTPVKKKAKKAGATAGKRKASGPPVSELITKAVAASKERSGVSLAALKKALAAAGYDVEKNNSRIKLGLKSLVSKGTLVQTKGTGASGSFKLNKKAASGEGKPKAKKAGAAKPRKPAGAAKKPKKVAGAATPKKSIKKTPKKVKKPATAAGTKKVAKSAKKVKTPQPKKAAKSPAKAKAPKPKAAKPKSGKPKVTKAKKAAPKKK**

**>P16615**

**MENAHTKTVEEVLGHFGVNESTGLSLEQVKKLKERWGSNELPAEEGKTLLELVIEQFEDLLVRILLLAACISFVLAWFEEGEETITAFVEPFVILLILVANAIVGVWQERNAENAIEALKEYEPEMGKVYRQDRKSVQRIKAKDIVPGDIVEIAVGDKVPADIRLTSIKSTTLRVDQSILTGESVSVIKHTDPVPDPRAVNQDKKNMLFSGTNIAAGKAMGVVVATGVNTEIGKIRDEMVATEQERTPLQQKLDEFGEQLSKVISLICIAVWIINIGHFNDPVHGGSWIRGAIYYFKIAVALAVAAIPEGLPAVITTCLALGTRRMAKKNAIVRSLPSVETLGCTSVICSDKTGTLTTNQMSVCRMFILDRVEGDTCSLNEFTITGSTYAPIGEVHKDDKPVNCHQYDGLVELATICALCNDSALDYNEAKGVYEKVGEATETALTCLVEKMNVFDTELKGLSKIERANACNSVIKQLMKKEFTLEFSRDRKSMSVYCTPNKPSRTSMSKMFVKGAPEGVIDRCTHIRVGSTKVPMTSGVKQKIMSVIREWGSGSDTLRCLALATHDNPLRREEMHLEDSANFIKYETNLTFVGCVGMLDPPRIEVASSVKLCRQAGIRVIMITGDNKGTAVAICRRIGIFGQDEDVTSKAFTGREFDELNPSAQRDACLNARCFARVEPSHKSKIVEFLQSFDEITAMTGDGVNDAPALKKAEIGIAMGSGTAVAKTASEMVLADDNFSTIVAAVEEGRAIYNNMKQFIRYLISSNVGEVVCIFLTAALGFPEALIPVQLLWVNLVTDGLPATALGFNPPDLDIMNKPPRNPKEPLISGWLFFRYLAIGCYVGAATVGAAAWWFIAADGGPRVSFYQLSHFLQCKEDNPDFEGVDCAIFESPYPMTMALSVLVTIEMCNALNSLSENQSLLRMPPWENIWLVGSICLSMSLHFLILYVEPLPLIFQITPLNVTQWLMVLKISLPVILMDETLKFVARNYLEPGKECVQPATKSCSFSACTDGISWPFVLLIMPLVIWVYSTDTNFSDMFWS**

**>P29400**

**MKLRGVSLAAGLFLLALSLWGQPAEAAACYGCSPGSKCDCSGIKGEKGERGFPGLEGHPGLPGFPGPEGPPGPRGQKGDDGIPGPPGPKGIRGPPGLPGFPGTPGLPGMPGHDGAPGPQGIPGCNGTKGERGFPGSPGFPGLQGPPGPPGIPGMKGEPGSIIMSSLPGPKGNPGYPGPPGIQGLPGPTGIPGPIGPPGPPGLMGPPGPPGLPGPKGNMGLNFQGPKGEKGEQGLQGPPGPPGQISEQKRPIDVEFQKGDQGLPGDRGPPGPPGIRGPPGPPGGEKGEKGEQGEPGKRGKPGKDGENGQPGIPGLPGDPGYPGEPGRDGEKGQKGDTGPPGPPGLVIPRPGTGITIGEKGNIGLPGLPGEKGERGFPGIQGPPGLPGPPGAAVMGPPGPPGFPGERGQKGDEGPPGISIPGPPGLDGQPGAPGLPGPPGPAGPHIPPSDEICEPGPPGPPGSPGDKGLQGEQGVKGDKGDTCFNCIGTGISGPPGQPGLPGLPGPPGSLGFPGQKGEKGQAGATGPKGLPGIPGAPGAPGFPGSKGEPGDILTFPGMKGDKGELGSPGAPGLPGLPGTPGQDGLPGLPGPKGEPGGITFKGERGPPGNPGLPGLPGNIGPMGPPGFGPPGPVGEKGIQGVAGNPGQPGIPGPKGDPGQTITQPGKPGLPGNPGRDGDVGLPGDPGLPGQPGLPGIPGSKGEPGIPGIGLPGPPGPKGFPGIPGPPGAPGTPGRIGLEGPPGPPGFPGPKGEPGFALPGPPGPPGLPGFKGALGPKGDRGFPGPPGPPGRTGLDGLPGPKGDVGPNGQPGPMGPPGLPGIGVQGPPGPPGIPGPIGQPGLHGIPGEKGDPGPPGLDVPGPPGERGSPGIPGAPGPIGPPGSPGLPGKAGASGFPGTKGEMGMMGPPGPPGPLGIPGRSGVPGLKGDDGLQGQPGLPGPTGEKGSKGEPGLPGPPGPMDPNLLGSKGEKGEPGLPGIPGVSGPKGYQGLPGDPGQPGLSGQPGLPGPPGPKGNPGLPGQPGLIGPPGLKGTIGDMGFPGPQGVEGPPGPSGVPGQPGSPGLPGQKGDKGDPGISSIGLPGLPGPKGEPGLPGYPGNPGIKGSVGDPGLPGLPGTPGAKGQPGLPGFPGTPGPPGPKGISGPPGNPGLPGEPGPVGGGGHPGQPGPPGEKGKPGQDGIPGPAGQKGEPGQPGFGNPGPPGLPGLSGQKGDGGLPGIPGNPGLPGPKGEPGFHGFPGVQGPPGPPGSPGPALEGPKGNPGPQGPPGRPGLPGPEGPPGLPGNGGIKGEKGNPGQPGLPGLPGLKGDQGPPGLQGNPGRPGLNGMKGDPGLPGVPGFPGMKGPSGVPGSAGPEGEPGLIGPPGPPGLPGPSGQSIIIKGDAGPPGIPGQPGLKGLPGPQGPQGLPGPTGPPGDPGRNGLPGFDGAGGRKGDPGLPGQPGTRGLDGPPGPDGLQGPPGPPGTSSVAHGFLITRHSQTTDAPQCPQGTLQVYEGFSLLYVQGNKRAHGQDLGTAGSCLRRFSTMPFMFCNINNVCNFASRNDYSYWLSTPEPMPMSMQPLKGQSIQPFISRCAVCEAPAVVIAVHSQTIQIPHCPQGWDSLWIGYSFMMHTSAGAEGSGQALASPGSCLEEFRSAPFIECHGRGTCNYYANSYSFWLATVDVSDMFSKPQSETLKAGDLRTRISRCQVCMKRT**

**>P43652**

**MKLLKLTGFIFFLFFLTESLTLPTQPRDIENFNSTQKFIEDNIEYITIIAFAQYVQEATFEEMEKLVKDMVEYKDRCMADKTLPECSKLPNNVLQEKICAMEGLPQKHNFSHCCSKVDAQRRLCFFYNKKSDVGFLPPFPTLDPEEKCQAYESNRESLLNHFLYEVARRNPFVFAPTLLTVAVHFEEVAKSCCEEQNKVNCLQTRAIPVTQYLKAFSSYQKHVCGALLKFGTKVVHFIYIAILSQKFPKIEFKELISLVEDVSSNYDGCCEGDVVQCIRDTSKVMNHICSKQDSISSKIKECCEKKIPERGQCIINSNKDDRPKDLSLREGKFTDSENVCQERDADPDTFFAKFTFEYSRRHPDLSIPELLRIVQIYKDLLRNCCNTENPPGCYRYAEDKFNETTEKSLKMVQQECKHFQNLGKDGLKYHYLIRLTKIAPQLSTEELVSLGEKMVTAFTTCCTLSEEFACVDNLADLVFGELCGVNENRTINPAVDHCCKTNFAFRRPCFESLKADKTYVPPPFSQDLFTFHADMCQSQNEELQRKTDRFLVNLVKLKHELTDEELQSLFTNFANVVDKCCKAESPEVCFNEESPKIGN**

**>P51635**

**MTASSVLLHTGQKMPLIGLGTWKSEPGQVKAAIKYALSVGYRHIDCASVYGNETEIGEALKESVGAGKAVPREELFVTSKLWNTKHHPEDVEPAVRKTLADLQLEYLDLYLMHWPYAFERGDNPFPKNADGTVKYDSTHYKETWKALEALVAKGLVKALGLSNFSSRQIDDVLSVASVRPAVLQVECHPYLAQNELIAHCQARGLEVTAYSPLGSSDRAWRHPDEPVLLEEPVVLALAEKHGRSPAQILLRWQVQRKVICIPKSITPSRILQNIQVFDFTFSPEEMKQLDALNKNWRYIVPMITVDGKRVPRDAGHPLYPFNDPY**

**>P54071**

**MAGYLRAVSSLCRASGSARTWAPAALTVPSWPEQPRRHYAEKRIKVEKPVVEMDGDEMTRIIWQFIKEKLILPHVDVQLKYFDLGLPNRDQTNDQVTIDSALATQKYSVAVKCATITPDEARVEEFKLKKMWKSPNGTIRNILGGTVFREPIICKNIPRLVPGWTKPITIGRHAHGDQYKATDFVVDRAGTFKLVFTPKDGSSAKEWEVYNFPAGGVGMGMYNTDESISGFAHSCFQYSIQKKWPLYLSTKNTILKAYDGRFKDIFQEIFDKHYKTDFDKNKIWYEHRLIDDMVAQVLKSSGGFVWACKNYDGDVQSDILAQGFGSLGLMTSVLVCPDGKTIEAEAAHGTVTRHYREHQKGRPTSTNPIASIFAWTRGLEHRGKLDGNQDLIRFAQTLEKVCVQTVESGAMTKDLAGCIHGLSNVKLNEHFLNTTDFLDTIKSNLDRALGKQ**

**>P61769**

**MSRSVALAVLALLSLSGLEAIQRTPKIQVYSRHPAENGKSNFLNCYVSGFHPSDIEVDLLKNGERIEKVEHSDLSFSKDWSFYLLYYTEFTPTEKDEYACRVNHVTLSQPKIVKWDRDM**

**>P61823**

**MALKSLVLLSLLVLVLLLVRVQPSLGKETAAAKFERQHMDSSTSAASSSNYCNQMMKSRNLTKDRCKPVNTFVHESLADVQAVCSQKNVACKNGQTNCYQSYSTMSITDCRETGSSKYPNCAYKTTQANKHIIVACEGNPYVPVHFDASV**

**>P68871**

**MVHLTPEEKSAVTALWGKVNVDEVGGEALGRLLVVYPWTQRFFESFGDLSTPDAVMGNPKVKAHGKKVLGAFSDGLAHLDNLKGTFATLSELHCDKLHVDPENFRLLGNVLVCVLAHHFGKEFTPPVQAAYQKVVAGVANALAHKYH**

**>P69905**

**MVLSPADKTNVKAAWGKVGAHAGEYGAEALERMFLSFPTTKTYFPHFDLSHGSAQVKGHGKKVADALTNAVAHVDDMPNALSALSDLHAHKLRVDPVNFKLLSHCLLVTLAAHLPAEFTPAVHASLDKFLASVSTVLTSKYR**

**>Q03626**

**MKKNREAQLCLFSALLAFLPFASLLNGNSKYMVLVPSQLYTETPEKICLHLYHLNETVTVTASLISQRGTRKLFDELVVDKDLFHCLSFTIPRLPSSEEEESLDINIEGAKHKFSERRVVLVKNKESVVFVQTDKPVYKPGQSVKFRVVSMDKNLHPLNELFPLAYIEDPKMNRIMQWQDIKTENGLKQLSFSLSAEPIQGPYKIVILKQSGVKEEHSFTVMEFVLPRFGVDVKVPNAISVYDEIINVTACAIYTYGKPVPGHVKISLCHGNPSFSSETKSACKEEDSELDNNGCSTQEVNITEFQLKENYLKMHQAFHVNATVTEEGTGSEFSGSGRIEVERTRNKFLFLKADSHFRHGIPFFVKIRLVDIKGDPIPNEQVFIKAQEAGYTNATTTDQHGLAKFSIDTSSISGYSLNIKVYHKEESSCIHSSCTAERHAEEHHTAYAVYSLSKSYIYLDTEAGVLPCNQIHTVQAHFILKGQVLGVLPQIVFHYLVMAQGSILQTGNHTHQVEPGVSQVQGNFALEIPVEFSMVPVAKMLIYTILPDGEVIADSVTFQVEKCLRNKVHLSFSPSQSLPASQTHMRVTASPQSLCGLRAVDQSVLLLKPEAELSPSLIYDLPGMQDSNFIPSSYHPFEDEYDCLMYQPRDTEELTYSVPYGREKDVYRYVRDMGLTAFTNLKIKHPTYCYEMNMVVLSAPAVESELSPRGGEFEMMPLGVNKSPLPKEPPRKDPPPKDPVIETIRNYFPETWIWDLVTVNSSGVTEVEMTVPDTITEWKAGALCLSNDTGLGLSSVATLQAFQPFFVELTMPYSVIRGEAFMLKATVMNYLPTSLPMAVQLEASPDFTAVPVGNDQDSYCLGANGRHTSSWLVTPKSLGNVNFSVSVEAQQSPELCGSQVATVPETGRKDTVVKVLIVEPEGIKKEHTFSSLLCASDAELSETLSLLLPPTVVKDSARAHFSVMGDILSSAIKNTQNLIQMPYGCGEQNMVLFAPNIYVLKYLNETQQLTEKIKSKALGYLRAGYQRELNYKHKDGSYSAFGDHNGQGQGNTWLTAFVLKSFAQARAFIFIDESHITDAFTWLSKQQKDSGCFRSSGSLFNNAMKGGVDDEITLSAYITMALLESSLPDTDPVVSKALGCLEASWETIEQGRNGSFVYTKTLMAYAFALAGNQEKRNEILKSLDKEAIREDNSIHWERPQKPTKSEGYLYTPQASSAEVEMSAYVVLARLTAQPAPSPEDLALSMGTIKWLTKQQNSHGGFSSTQDTVVALDALSKYGAATFSKSQKTPLVTIQSSGSFSQKFQVDNSNRLLLQQVSLPDIPGNYTVSVSGEGCVYAQTTLRYNMPLEKQQPAFALKVQTVPLTCNNPKGQNSFQISLEISYTGSRPASNMVIADVKMLSGFIPLKPTVKKLERLEHVSRTEVTTNNVLLYLDQVTNQTLSFSFIIQQDIPVKNLQPAIVKVYDYYETDEVAFAEYSSPCSSDKQNV**

**>Q16778**

**MPEPAKSAPAPKKGSKKAVTKAQKKDGKKRKRSRKESYSIYVYKVLKQVHPDTGISSKAMGIMNSFVNDIFERIAGEASRLAHYNKRSTITSREIQTAVRLLLPGELAKHAVSEGTKAVTKYTSSK**

**>Q6PIK1**

**MAWALLLLTLLTQGTGSWAQSALTQPPSASGSPGQSVTISCTGTSSDVGGYNYVSWYQQHPGKAPKLMIYEVNKRPSGVPDRFSGSKSGNTASLTVSGLQAEDEADYYCSSYAGSNNYVFGTGTKVTVLGQPKANPTVTLFPPSSEELQANKATLVCLISDFYPGAVTVAWKADGSPVKAGVETTKPSKQSNNKYAASSYLSLTPEQWKSHRSYSCQVTHEGSTVEKTVAPTECS**

**>Q6PIL0**

**MDWTWSILFLVAAATGTYSQVQLVQSGHEVKQPGASVKVSCKASGYSFTTYGMNWVPQAPGQGLEWMGWFNTYTGNPTYAQGFTGRFVFSMDTSASTAYLQISSLKAEDMAMYYCARYTMWKPTS**

**>Q86XX4**

**MGVLKVWLGLALALAEFAVLPHHSEGACVYQDSLLADATIWKPDSCQSCRCHGDIVICKPAVCRNPQCAFEKGEVLQIAANQCCPECVLRTPGSCHHEKKIHEHGTEWASSPCSVCSCNHGEVRCTPQPCPPLSCGHQELAFIPEGSCCPVCVGLGKPCSYEGHVFQDGEDWRLSRCAKCLCRNGVAQCFTAQCQPLFCNQDETVVRVPGKCCPQCSARSCSAAGQVYEHGEQWSENACTTCICDRGEVRCHKQACLPLRCGKGQSRARRHGQCCEECVSPAGSCSYDGVVRYQDEMWKGSACEFCMCDHGQVTCQTGECAKVECARDEELIHLDGKCCPECISRNGYCVYEETGEFMSSNASEVKRIPEGEKWEDGPCKVCECRGAQVTCYEPSCPPCPVGTLALEVKGQCCPDCTSVHCHPDCLTCSQSPDHCDLCQDPTKLLQNGWCVHSCGLGFYQAGSLCLACQPQCSTCTSGLECSSCQPPLLMRHGQCVPTCGDGFYQDRHSCAVCHESCAGCWGPTEKHCLACRDPLHVLRDGGCESSCGKGFYNRQGTCSACDQSCDSCGPSSPRCLTCTEKTVLHDGKCMSECPGGYYADATGRCKVCHNSCASCSGPTPSHCTACSPPKALRQGHCLPRCGEGFYSDHGVCKACHSSCLACMGPAPSHCTGCKKPEEGLQVEQLSDVGIPSGECLAQCRAHFYLESTGICEACHQSCFRCAGKSPHNCTDCGPSHVLLDGQCLSQCPDGYFHQEGSCTECHPTCRQCHGPLESDCISCYPHISLTNGNCRTSCREEQFLNLVGYCADCHHLCQHCAADLHNTGSICLRCQNAHYLLLGDHCVPDCPSGYYAERGACKKCHSSCRTCQGRGPFSCSSCDTNLVLSHTGTCSTTCFPGHYLDDNHVCQPCNTHCGSCDSQASCTSCRDPNKVLLFGECQYESCAPQYYLDFSTNTCKECDWSCSACSGPLKTDCLQCMDGYVLQDGACVEQCLSSFYQDSGLCKNCDSYCLQCQGPHECTRCKGPFLLLEAQCVQECGKGYFADHAKHKCTACPQGCLQCSHRDRCHLCDHGFFLKSGLCVYNCVPGFSVHTSNETCSGKIHTPSLHVNGSLILPIGSIKPLDFSLLNVQDQEGRVEDLLFHVVSTPTNGQLVLSRNGKEVQLDKAGRFSWKDVNEKKVRFVHSKEKLRKGYLFLKISDQQFFSEPQLINIQAFSTQAPYVLRNEVLHISRGERATITTQMLDIRDDDNPQDVVIEIIDPPLHGQLLQTLQSPATPIYQFQLDELSRGLLHYAHDGSDSTSDVAVLQANDGHSFHNILFQVKTVPQNDRGLQLVANSMVWVPEGGMLQITNRILQAEAPGASAEEIIYKITQDYPQFGEVVLLVNMPADSPADEGQHLPDGRTATPTSTFTQQDINEGIVWYRHSGAPAQSDSFRFEVSSASNAQTRLESHMFNIAILPQTPEAPKVSLEASLHMTAREDGLTVIQPHSLSFINSEKPSGKIVYNITLPLHPNQGIIEHRDHPHSPIRYFTQEDINQGKVMYRPPPAAPHLQELMAFSFAGLPESVKFHFTVSDGEHTSPEMVLTIHLLPSDQQLPVFQVTAPRLAVSPGGSTSVGLQVVVRDAETAPKELFFELRRPPQHGVLLKHTAEFRRPMATGDTFTYEDVEKNALQYIHDGSSTREDSMEISVTDGLTVTMLEVRVEVSLSEDRGPRLAAGSSLSITVASKSTAIITRSHLAYVDDSSPDPEIWIQLNYLPSYGTLLRISGSEVEELSEVSNFTMEDINNKKIRYSAVFETDGHLVTDSFYFSVSDMDHNHLDNQIFTIMITPAENPPPVIAFADLITVDEGGRAPLSFHHFFATDDDDNLQRDAIIKLSALPKYGCIENTGTGDRFGPETASDLEASFPIQDVLENYIYYFQSVHESIEPTHDIFSFYVSDGTSRSEIHSINITIERKNDEPPRMTLQPLRVQLSSGVVISNSSLSLQDLDTPDNELIFVLTKKPDHGHVLWRQTASEPLENGRVLVQGSTFTYQDILAGLVGYVPSVPGMVVDEFQFSLTDGLHVDTGRMKIYTELPASDTPHLAINQGLQLSAGSVARITEQHLKVTDIDSDDHQVMYIMKEDPGAGRLQMMKHGNLEQISIKGPIRSFTQADISQGQPEYSHGTGEPGGSFAFKFDVVDGEGNRLIDKSFSISISEDKSPPVITTNKGLVLDENSVKKITTLQLSATDQDSGPTELIYRITRQPQLGHLEHAASPGIQISSFTQADLTSRNVQYVHSSEAEKHSDAFSFTLSDGVSEVTQTFHITLHPVDDSLPVVQNLGMRVQEGMRKTITEFELKAVDADTEAESVTFTIVQPPRHGTIERTSNGQHFHLTSTFTMKDIYQNRVSYSHDGSNSLKDRFTFTVSDGTNPFFIIEEGGKEIMTAAPQPFRVDILPVDDGTPRIVTNLGLQWLEYMDGKATNLITKKELLTMDPDTEDAQLVYEITTGPKHGFVENKLQPGRAAATFTQEDVNLGLIRYVLHKEKIREMMDSFQFLVKDSKPNVVSDNVFHIQWSLISFKYTSYNVSEKAGSVSVTVQRTGNLNQYAIVLCRTEQGTASSSSQPGQQDYVEYAGQVQFDEREDTKSCTIVINDDDVFENVESFTVELSMPAYALLGEFTQAKVIINDTEDEPTLEFDKKIYWVNESAGFLFAPIERKGDASSIVSAICYTVPKSAMGSLFYALESGSDFKSRGMSAASRVIFGPGVTMSTCDVMLIDDSEYEEEEEFEIALADASDNARIGRVATAKVLISGPNDASTVSLGNTAFTVSEDAGTVKIPVIRHGTDLSTFASVWCATRPSDPASATPGVDYVPSSRKVEFGPGVIEQYCTLTILDDTQYPVIEGLETFVVFLSSAQGAELTKPFQAVIAINDTFQDVPSMQFAKDLLLVKEKEGVLHVPITRSGDLSYESSVRCYTQSHSAQVMEDFEERQNADSSRITFLKGDKVKNCTVYIHDDSMFEPEEQFRVYLGLPLGNHWSGARIGKNNMATITISNDEDAPTIEFEEAAYQVREPAGPDAIAILNIKVIRRGDQNRTSKVRCSTRDGSAQSGVDYYPKSRVLKFSPGVDHIFFKVEILSNEDREWHESFSLVLGPDDPVEAVLGDVTTATVTILDQEAAGSLILPAPPIVVTLADYDHVEEVTKEGVKKSPSPGYPLVCVTPCDPHFPRYAVMKERCSEAGINQTSVQFSWEVAAPTDGNGARSPFETITDNTPFTSVNHMVLDSIYFSRRFHVRCVAKAVDKVGHVGTPLRSNIVTIGTDSAICHTPVVAGTSRGFQAQSFIATLKYLDVKHKEHPNRIHISVQIPHQDGMLPLISTMPLHNLHFLLSESIYRHQHVCSNLVTTYDLRGLAEAGFLDDVVYDSTALGPGYDRPFQFDPSVREPKTIQLYKHLNLKSCVWTFDAYYDMTELIDVCGGSVTADFQVRDSAQSFLTVHVPLYVSYIYVTAPRGWASLEHHTEMEFSFFYDTVLWRTGIQTDSVLSARLQIIRIYIREDGRLVIEFKTHAKFRGQFVMEHHTLPEVKSFVLTPDHLGGIEFDLQLLWSAQTFDSPHQLWRATSSYNRKDYSGEYTIYLIPCTVQPTQPWVDPGEKPLACTAHAPERFLIPIAFQQTNRPVPVVYSLNTEFQLCNNEKVFLMDPNTSDMSLAEMDYKGAFSKGQILYGRVLWNPEQNLNSAYKLQLEKVYLCTGKDGYVPFFDPTGTIYNEGPQYGCIQPNKHLKHRFLLLDRNQPEVTDKYFHDVPFEAHFASELPDFHVVSNMPGVDGFTLKVDALYKVEAGHQWYLQVIYIIGPDTISGPRVQRSLTAPLRRNRRDLVEPDGQLILDDSLIYDNEGDQVKNGTNMKSLNLEMQELAVAASLSQTGASIGSALAAIMLLLLVFLVACFINRKCQKQRKKKPAEDILEEYPLNTKVEVPKRHPDRVEKNVNRHYCTVRNVNILSEPEAAYTFKGAKVKRLNLEVRVHNNLQDGTEV**

**>Q8NF17**

**LWLWGREWAKVTQVAPARRTPNAREPRHWTLPGPSWIDKNQGASAPWAQLCPTPQSHGAISLAASTKGPSVFPLAPCSRSTSGGTAALGCLVKDYFPEPVTVSWNSGALTSGVHTFPAVLQSSGLYSLSSVVTVPSSSLGTQTYTCNVNHKPSNTKVDKRVELKTPLGDTTHTCPRCPEPKSCDTPPPCPRCPEPKSCDTPPPCPRCPEPKSCDTPPPCPRCPAPELLGGPSVFLFPPKPKDTLMISRTPEVTCVVVDVSHEDPEVQFKWYVDGVEVHNAKTKPREEQFNSTFRVVSVLTVLHQDWLNGKEYKCKVSNKALPAPIEKTISKTKGQPREPQVYTLPPSREEMTKNQVSLTCLVKGFYPSDIAVEWESSGQPENNYNTTPPMLDSDGSFFLYSKLTVDKSRWQQGNIFSCSVMHEALHNRFTQKSLSLSPELQLEESCAEAQDGELDGLWTTITIFITLFLLSVCYSATVTFFKVKWIFSSVVDLKQTIIPDYRNMIGQGA**

**>Q9ULR0**

**MARNAEKAMTALARFRQAQLEEGKVKERRPFLASECTELPKAEKWRRQIIGEISKKVAQIQNAGLGEFRIRDLNDEINKLLREKGHWEVRIKELGGPDYGKVGPKMLDHEGKEVPGNRGYKYFGAAKDLPGVRELFEKEPLPPPRKTRAELMKAIDFEYYGYLDEDDGVIVPLEQEYEKKLRAELVEKWKAEREARLARGEKEEEEEEEEEINIYAVTEEESDEEGSQEKGGDDSQQKFIAHVPVPSQQEIEEALVRRKKMELLQKYASETLQAQSEEARRLLGY**
